# Towards the ground state of molecules via diffusion Monte Carlo on neural networks

**DOI:** 10.1038/s41467-023-37609-3

**Published:** 2023-04-03

**Authors:** Weiluo Ren, Weizhong Fu, Xiaojie Wu, Ji Chen

**Affiliations:** 1 ByteDance Research, Zhonghang Plaza, No. 43, North 3rd Ring West Road, Haidian District Beijing, People’s Republic of China; 2 School of Physics, Peking University, 100871 Beijing, People’s Republic of China; 3 Interdisciplinary Institute of Light-Element Quantum Materials, Frontiers Science Center for Nano-Optoelectronics, Peking University, 100871 Beijing, People’s Republic of China

**Keywords:** Chemical physics, Physical chemistry, Computational chemistry

## Abstract

Diffusion Monte Carlo (DMC) based on fixed-node approximation has enjoyed significant developments in the past decades and become one of the go-to methods when accurate ground state energy of molecules and materials is needed. However, the inaccurate nodal structure hinders the application of DMC for more challenging electronic correlation problems. In this work, we apply the neural-network based trial wavefunction in fixed-node DMC, which allows accurate calculations of a broad range of atomic and molecular systems of different electronic characteristics. Our method is superior in both accuracy and efficiency compared to state-of-the-art neural network methods using variational Monte Carlo (VMC). We also introduce an extrapolation scheme based on the empirical linearity between VMC and DMC energies, and significantly improve our binding energy calculation. Overall, this computational framework provides a benchmark for accurate solutions of correlated electronic wavefunction and also sheds light on the chemical understanding of molecules.

## Abstract (web summary)

An accurate ab initio calculation of molecules is fundamental to chemical and physical sciences. Here, the authors integrate a neural-network wavefunction into the fixed-node diffusion Monte Carlo, resulting in accurate calculations of a diverse range of systems, offering insights into complex many-body electronic wave functions.

## Introduction

Since the establishment of quantum wavefunction theory by Erwin Schrödinger, ab initio electronic structure calculation has become one of the holy grails in chemistry^1,2^. Molecules generally consist of a set of nuclei bonded together via electrons through electrostatic interactions. Therefore, the ground state electronic structure, i.e., the many-body electronic wavefunction, is very much the most fundamental property, based on which we form the basic understanding of molecules. On top of the ground state wavefunction solution, one may further study electronic excitation, calculate nuclear forces and vibrations, optimize molecular structures, model dynamics and reactions, etc.^3^. Approximated methods, such as density functional theory and post Hartree-Fock methods have been widely employed for these purposes, but challenges still exist when high accuracy is needed^4,5^. For instance, the sub-chemical-accuracy is often desired to predict adsorption of molecules on surfaces, the packing order of organic chemicals, and the hydrogen bonding of water and biological molecules^6,7^. Therefore, pushing the limit towards the exact ground state wavefunction of molecules is of both fundamental importance and practical relevance.

Stochastic approaches, i.e., quantum Monte Carlo (QMC) methods, have been a competitive rival of the deterministic methods in chasing the ground truth of many-body electronic wavefunction of molecules^8–11^. In particular, diffusion Monte Carlo (DMC), an approach based on ground state projection, is capable of treating dynamic correlations and reaching sub-chemical-accuracy for molecules^12,13^. However, effective DMC algorithms usually work together with the so-called fixed-node approximation^14,15^, and the accuracy is only assured when a good trial wavefunction containing the correct nodal structure is provided in advance^16^. Despite many progresses have been made to improve the trial wavefunction, e.g., using physically more meaningful ansatz or combined with multi-determinant post Hartree-Fock wavefunctions^13,17,18^, the fixed-node approximation remains as the Achilles’ heel of DMC.

Recently, it has been shown that machine learning techniques such as neural networks can lend strong support to describe the electronic structure of molecular systems and provide a powerful way to reconstruct the many-body wavefunction^19–26^. FermiNet is one of the notable examples, which has already shown promising results for small molecules consisting of typically less than 30 electrons^20,21,27^. In these neural network wavefunction methods, variational Monte Carlo (VMC) is often employed to train the network on the fly. Despite its effectiveness on small molecules, it remains to be challenging to apply neural network-based VMC on larger systems, due to required large computation resources and long converging time.

In this work, we integrate the FermiNet neural network wavefunction into DMC. This approach takes advantage of the accurate trial wavefunction of FermiNet and the efficient ground state projection of DMC, which allows calculations of a range of systems to unprecedented accuracy. We refer to the vanilla FermiNet approach as FermiNet-VMC, and refer to our FermiNet-based DMC approach as FermiNet-DMC. Compared to FermiNet-VMC, FermiNet-DMC is able to achieve lower variational ground state energy at reduced computational cost. We carry out tests on atoms as well as molecules including N_2_, cyclobutadiene, water dimer, benzene and benzene dimer. We also present the empirical linear relation between VMC and DMC energies in our calculations and introduce an extrapolation scheme accordingly. Insights to the electronic structure of these systems obtained from our calculations are also discussed.

## Results

### Computational framework

**Fig. 1:**
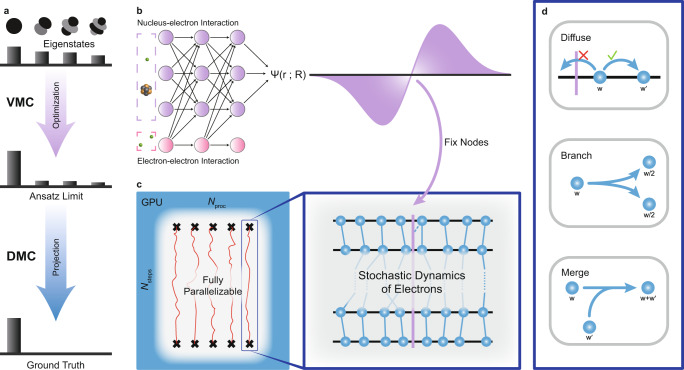
Computational framework. **a** A sketch of a brief overview on variational Monte Carlo (VMC) and diffusion Monte Carlo (DMC) from the perspective of eigenstates composition. Atomic orbitals represent different eigenstates, and histograms indicate the weight of each eigenstate in the state decomposition. Top: A randomly initialized state with no dominant eigenstate. Middle: The output state of VMC where the ground state dominates, but other eigenstates are still non-negligible due to ansatz limitations. Bottom: The output state of DMC, which surpasses ansatz limitations and reaches the ground state. **b** Left: a neural network ansatz of wavefunction; right: one dimensional projection of a many-electron wavefunction and its nodal surface. **c** Left: parallelized diffusion Monte Carlo processes on GPU; right: zoom in to the stochastic dynamics of each walker containing configurations of all electrons in the system, while the nodal structure is fixed. This panel is inspired by and adapted with permission from the website of Quantum Monte Carlo for Chemistry @ Toulouse (http://qmcchem.ups-tlse.fr/index.php/Quantum_Monte_Carlo_for_Chemistry_@_Toulouse)^56^. **d** Three key steps in diffusion Monte Carlo. Each walker is assigned with a weight, which evolves every iteration. Diffuse: The stochastic propagation of walkers without crossing the nodal surface. Branch: Split one walker when its weight becomes too large. Merge: Merge two walkers when their weights become too small.

As illustrated in Fig. 1a, in the traditional electronic structure approach, diffusion Monte Carlo is often used after optimization of trial wavefunction using VMC, which approaches the limit of a given wavefunction ansatz. DMC further purifies the true ground state out of other contaminating eigenstates, and it often allows the breaking through of the ansatz limit. However, to overcome the notorious sign problem, nodal points where the wavefunction is zero have to be fixed in DMC, and walkers are only allowed to evolve in each fixed nodal pocket. Here, the idea is to implement the recently developed neural network as an accurate wavefunction ansatz (Fig. 1b). On one hand, the wavefunction learned by the neural network automatically reproduces an accurate representation of the mysterious nodal structure of many electrons of molecules. The accurate nodal structure ensures that the subsequent DMC simulation with fixed nodes does not yield bias to the ground state. On the other hand, compared with neural networks-based VMC, our scheme only requires the information of the nodal structure instead of the full wavefunction. It is reasonable to expect the nodal structure to be simpler characterized than the full wavefunction.

Our multi-walker DMC algorithm is implemented in a fully parallel manner, in which each walker independently simulates the stochastic dynamics of electrons (Fig. 1c). The three key steps in our DMC algorithm are diffusion, branching, and merging (Fig. 1d), and they ensure that the equilibrium is reached for each walker after simulation in terms of the probability distribution of different electronic configurations. The diffusion step changes the configuration of electrons from one to another, while the cross-node movement is forbidden. Branching and merging control the total population of walkers during the simulation. In this work, we have implemented a GPU and neural network friendly DMC algorithm, which can be easily scaled out to multiple computing nodes. The runtime for one step in FermiNet-DMC is almost identical to that in FermiNet-VMC. Therefore, to compare the efficiency or total runtime between FermiNet-DMC and FermiNet-VMC, we only need to compare the number of steps in those processes. More methodological and technical details are provided in the “Methods” section and the Supplementary Notes 1–5.

### Single atoms

**Fig. 2:**
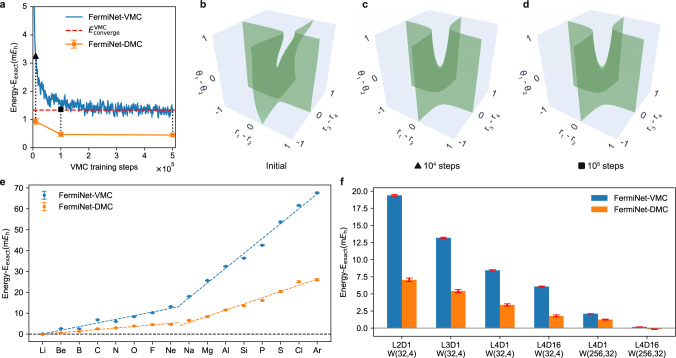
Accuracy and efficiency of FermiNet-DMC on single atoms. **a** The blue line shows the energy of a 2-layer FermiNet as a function of training iteration for a Be atom. The orange symbols show the DMC energy obtained with the trial wavefunction at the 10^4^th, 10^5^th and 5 × 10^5^th training iteration, respectively. The red dashed line shows the final convergence of VMC energy. **b**–**d** The three isosurfaces show the three-dimensional cuts of the full 11D nodal structure obtained at different training iterations (see Methods, section “Nodal structure and wavefunction visualization for plotting details”). **e** The calculated energies of atoms with respect to the reference ground state energy as the number of electrons increases with a 2-layer network. The dashed lines are linear fittings on the second and the third period elements, respectively. **f** The energy error with different settings of FermiNet for a Ne atom. Three variables are respectively the number of layers (L), the number of determinants (D) and the width (W) of each layer. All the energy reference values are from Chakravorty et al.^57^.

Neural network models are faced with the trade-off between model expressiveness and computational intensiveness. For powerful models like FermiNet, it may take hundreds of thousands iterations to converge in the training process even for small benchmark systems with just a few electrons. Figure 2 shows calculations for single atoms with a shallow and narrow FermiNet ansatz with only 2 layers of rather small number of neurons (see Supplementary Table S3 for details). The network is designed to be restricted so that we can study FermiNet’s performance when it is not expressive enough for the considered systems. This situation is of practical importance especially when we are interested in applying neural network-based QMC methods to large systems of one hundred electrons or more. As shown in Fig. 2a, a common pattern of FermiNet’s training progress is that the energy curve drops to a fairly low level in a short amount of time and then slowly converges to its limit. Figure 2a is a calculation on the Be atom with the mentioned small network, and after 5 × 10^5^ steps of training, which ensures complete convergence, the systematic error still can not be reduced to within the chemical accuracy. In addition, the computational cost could scale up quickly for larger systems even on the most advanced modern computation platforms such as NVIDIA’s Tesla A100 GPU. This issue prevents accurate calculations for more than 30 correlated electrons^20,21^.

The combination of the FermiNet neural network wavefunction ansatz and DMC achieves a substantial improvement in both accuracy and efficiency. For Be atom and the same simple neural network, FermiNet-DMC energy drops to within 1 mHa with respect to the reference value of the total energy. The DMC data is obtained with 10^5^ steps of simulation, and the variance of DMC is also significantly reduced. It is also encouraging to see that even when we start from the trial wavefunction after 10^4^ steps of training, the DMC energy obtained subsequently is also converged within 1 mHa to the exact value. At the 10^5^ step when the training has not yet completely converged, the DMC energy is already consistent with the result obtained at the 5 × 10^5^ step. The good performance of DMC based on undertrained trial wavefunctions suggests the nodal structure is well characterized before the wavefunction is fully trained in the neural network ansatz. In Fig. 2b–d, we present the three-dimensional cuts of the full 11-dimensional (11D) nodal structure of the FermiNet wavefunction at the initial, 10^4^, and 10^5^ step. The 10^5^ step nodal structure is very well converged to the correct one obtained from CI calculations^28^, and the nodal structure at 10^4^ step is also qualitatively same, explaining the high accuracy obtained subsequently using DMC. For comparison, the nodal structure of the initial wavefunction is also shown. Because of the fact that only the nodal structure determines the accuracy of DMC, the training process of neural network functions can be significantly shortened. Overall, to reach chemical accuracy for Be atom, the cost of FermiNet-DMC is only a fraction of the cost of FermiNet-VMC.

Figure 2e further shows the energy of FermiNet-based VMC and DMC for different atoms in order of the number of electrons under the same 2-layer network. We try different learning rates and train enough iterations (10^6^ for S, Cl, Ar and 5 × 10^5^ for the other atoms) to ensure that we make full use of the expressive power of the network. As expected the error of VMC increases when the number of electrons in the system increases and the complexity of the system gradually exceeds the expressive limits of the neural network. With DMC the errors are reduced by more than half. The dashed lines are linear fittings of the VMC and DMC energy. And the deviation of the data points from the fitting curves indicates that there is a correlation between the DMC and the VMC energy: when the VMC energy is comparably better, the DMC error is also smaller. The linear rising of DMC error shows that the training of nodal structure also becomes increasingly difficult when system size increases, and the correlation between the VMC and the DMC error indicates the information of the nodal structure is closely entangled with the full wavefunction. Note that we use a 2-layer network here in order to examine the behavior of FermiNet VMC and DMC in the regime where the network ansatz is relatively restricted for the considered systems, while FermiNet-VMC can be more expressive to achieve high accuracy for those atoms with more layers and neurons, as shown in Pfau et al.^20^.

Moreover, the improvement of DMC suggests that it may take a smaller and hence more efficient network to represent the nodal surface, without affecting the DMC accuracy. In Fig. 2f, we present a set of such tests on Ne atom, where the complexity of the neural network is labeled as (L,D,W) to indicate the number of layers, the number of determinants, and the width of each layer in the network, respectively. Overall, when the expressiveness of the network is reduced both VMC and DMC are affected in terms of their accuracy. Therefore, all the calculations suggest that the VMC energy is a good indicator of not only how well the wavefunction is optimized but also the quality of its nodal structure. The behavior is also expected for other neural network wavefunction ansatz. Combined with the typical first-steep-then-flat optimization curve in neural networks, we can automate the switching-on of DMC and minimize the total cost of calculations at targeted accuracy.

Building upon the successful treatments of FermiNet-DMC on atoms, we now extend the approach to larger molecules.

### Nitrogen molecule

**Fig. 3:**
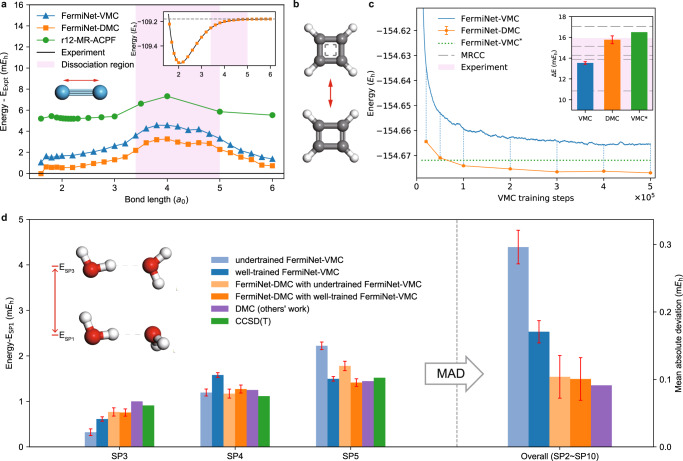
Calculations on N_2_, cyclobutadiene, and water dimer. **a** Main panel: calculated energy of N_2_ at different bond length, plotted as the difference to the experimental data^29^. For comparison, the green line is the highly accurate r12-MR-ACPF results under a modified basis set based on aug-cc-pV5Z^30^. Inset: the dissociation curves from experimental data (black line) and FermiNet-DMC (orange squares). The negligible error bars (less than 0.1 mHa) are not plotted. The pink backgrounds highlight the dissociation region where correlations are strong. **b** Molecular structures of cyclobutadiene’s equilibrium state (bottom) and transition state (top). **c** Main panel: the ground state energy of cyclobutadiene’s equilibrium state as a function of the VMC training step. FermiNet-DMC energy is calculated using the trial wavefunction at the corresponding training steps. FermiNet-VMC^*^ indicates the result from Spencer et al.^21^. Inset: the transition barrier of cyclobutadiene calculated with different methods. Pink background indicates the range of experimental estimates between 2.5 and 15.9 mHa, while we show only the part above 9 mHa to highlight differences between QMC results. Gray dashed lines indicate results from five multi-reference coupled cluster (MRCC) methods (top to bottom): MR-DI-EOMCCSD, RMRCCSD(T), Mk-MRCCSD(T), MRCISD+Q and BW-MRCCSD(T)^58^. **d** Left: three of the relative energies of the 10 Smith stationary points SP*n*(*n* = 1, 2, …, 10)^32^. For the other results, see Supplementary Fig. 6. The SP1 structure is the global minimum and is taken as the reference. The geometries of SP1 and SP3 are shown as insets while the others are included in Supplementary Fig. 4. Two neural networks have been trained for 10^5^ steps and 3 × 10^5^ steps, which are dubbed as “undertrained” and “well-trained''. All the geometries are optimized by CCSD(T)^34^. The CCSD(T) energies and the DMC results with the conventional Slater-Jastrow ansatz^35^ are also plotted for comparison. The error bar of the energy difference is calculated as the square root of the sum of the squares of each energy estimator’s standard error. Right: mean absolute deviation from CCSD(T) results over all the relative energies.

The first example is the dissociation curve of N_2_ molecule. At equilibrium N_2_ forms a strong triple covalent bond at 2.1 a.u., and the dissociation is accompanied by a severe bond breaking process, which is strongly correlated in nature. Therefore, the dissociation curve of N_2_ is often used to benchmark electronic structure methods’ description of strong correlation. In DMC, this is also highly relevant because the nodal structure is directly affected by electron correlation. Figure 3a plots the relative energy of N_2_ with respect to the experimental reference ^29^ as a function of the bond length. The results from FermiNet-VMC and r12-MR-ACPF, a state-of-the-art traditional multi-reference approach^30^, are also shown. We can see that our DMC calculations are consistently better than those references, with an error of less than 1 mHa in a wide range of bond length. The largest error comes, not surprisingly, around the dissociation point near 4 a.u., and yet the error is only 3 mHa. In fact, our results can be considered as the most accurate ab initio one of N_2_ dissociation curve reported so far. It is worth noting that the FermiNet-VMC results here have been remarkably accurate, whose deviation from experiment curve is within 2 mHa near equilibrium and 4 mHa in dissociation region. Yet our FermiNet-DMC results can improve averagely about 1 mHa. For comparison, CCSD(T) calculation (not plotted), which is known as the “golden standard” in quantum chemistry, have an error of 25 mHa around 4 a.u.^20^. In terms of relative energy, the non-parallelity error (NPE) of FermiNet-DMC (3.28 mHa) is only slightly better than that of FermiNet-VMC (3.53 mHa), consistent with mild improvement on small systems reported in Wilson et al.^31^, and both are comparable to the state-of-the-art r12-MR-ACPF result (2.14 mHa).

The remaining error source of DMC is the nodal structure error produced in the training of neural network using VMC, which is fully reflected on the shape of the FermiNet VMC and DMC curves. The results of FermiNet-DMC are close to the experimental fitting curve within 1 mHa outside the dissociation region and cannot go any lower due to the variational property. So, when combined with a more expressive or better trained neural network that can handle the dissociation region, it is very likely that the full dissociation curve of N_2_ can be reproduced by DMC within an error of 1 mHa, meaning that DMC can also solve strongly correlated systems within chemical accuracy.

### Cyclobutadiene

A similar example is the structural transition of cyclobutadiene, which is also well-known for its multi-referential nature. The neural network-based VMC models^21,22^ have already shown promising results on cyclobutadiene. FermiNet-DMC can handle this system with higher accuracy and reduced computational cost.

In our experiments, VMC process takes around 3 × 10^5^ steps to converge, though the converged result is still around 7 mHa higher than the reported value in Spencer et al.^21^, which converges in 2 × 10^5^ steps. This is probably because we use different training hyperparamters, or simply because our optimization process gets trapped in a bad local minimum. However, our final DMC result is around 4 mHa lower than the reference data^21^. This demonstrates the effectiveness of our DMC implementation as a seamless extension to VMC. Namely even if the optimization in VMC does not work well, the following DMC process can still bring the energy calculation to a highly accurate level. This is especially important for neural network-based VMC, because its optimization is significantly trickier to tune and requires a longer time to completely converge, compared to conventional VMC. Here, the DMC finite time-step error is negligible as illustrated in Supplementary Fig. 1, which guarantees the variational property of our FermiNet-DMC results,

With 10^5^ VMC and 10^5^ DMC steps, FermiNet-DMC’s energy result is 2 mHa lower than the reference data in Spencer et al.^21^ produced from a training phase with 2 × 10^5^ VMC steps. Note that in this case our number of total QMC steps is still slightly less than the ref. ^21^ due to the required inference phase in FermiNet-VMC. Therefore, FermiNet-DMC should be preferred for its lower variational energy at the same or less computational cost.

The automerization energy difference of cyclobutadiene is shown in the inset panel of Fig. 3c. Neural network-based VMC gives an accurate automerization energy difference of cyclobutadiene^21,22^. It is consistent with the high-end of the experimental data. The results of FermiNet-DMC are also in the same region. See Supplementary Note 9 for more details, including the training curve for transition configuration and the DMC energy data for both equilibrium and transition configurations.

### Water dimer

In addition to the strong covalent bonding, where static correlation is more essential, molecular systems with weaker hydrogen bonding and non-covalent interactions can also be challenging because of dynamic correlations. To this end, we have carried out FermiNet-DMC calculations on the 10 Smith stationary point of water dimer^32^. The 10 structures, as illustrated in Fig. 3d and Supplementary Fig. 4, have different hydrogen bonding configurations and their relative energies are used to benchmark the performance of electronic structure methods and force field models on hydrogen bonding systems^33^. With 10 total energy results (plotted in Supplementary Fig. 5) and 9 relative energy results (plotted in Supplementary Fig. 6), we can thus have a rather credible investigation on the error cancellation performance of FermiNet-VMC and FermiNet-DMC. We compare the energy results of FermiNet-DMC with an undertrained network and a well-trained network as trial wavefunctions respectively. The undertrained network is trained by VMC in 10^5^ steps, while the well-trained network is trained by VMC in 3 × 10^5^ steps. CCSD(T) results^34^ are displayed as benchmarks for their high accuracy for such type of systems.

As shown in Fig. 3d, the undertrained FermiNet-VMC performs badly on SP3 and SP5, and so does the well-trained FermiNet-VMC on SP4, though some of the FermiNet-VMC results are quite close to the benchmark results (e.g., SP7 and SP8 in Supplementary Fig. 6). On the other hand, FermiNet-DMC performs consistently well no matter which network is used as trial wavefunction, undertrained or well-trained. Overall, the mean absolute deviations from the benchmark CCSD(T) results are also given in Fig. 3d, from which we can clearly tell the improvement of FermiNet-DMC on relative energy calculations. For comparison purpose, we also show DMC results with traditional Slater-Jastrow wavefunction ansatz^35^, whose accuracy is at the same level with FermiNet-DMC as the difference is negligible compared to the statistical error. The inferior performance of FermiNet-VMC may be due to the different degree of convergence in different systems, while FermiNet-DMC provides a more efficient and practical solution than fully converged FermiNet-VMC.

### Benzene

**Fig. 4:**
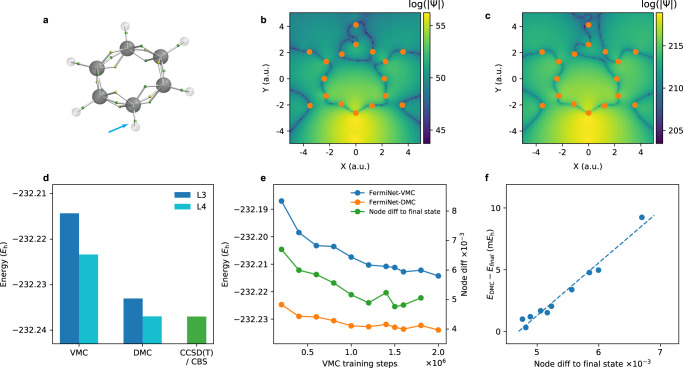
Calculations on benzene. **a** Atomic structure of the benzene molecule and representative electron positions^39^. Small balls represent electrons, with spin indicated by their colors, and larger balls represent nuclei. The electron pointed by the blue arrow is an arbitrarily chosen one for nodal set visualization in **b** and **c**. Rods are drawn to connect the nucleus with electrons nearby. **b** and **c** The log-scaled magnitude of unnormalized FermiNet wavefunctions for a benzene molecule. Each slice is generated by moving a single spin-up electron in the square [−5 a.u., 5 a.u.]^2^ on *X*–*Y* plane while fixing all other electrons in the representative positions shown in **a**. The dark curves are the nodes and the orange points are the fixed spin-up electrons projected onto *X*–*Y* plane. The moving electron for **b** and **c** corresponds to the one pointed by the blue arrow on the bottom C–H bond in **a**. **b** shows a slice for a 4-layer FermiNet while **c** is for a 3-layer FermiNet. **d** Ground state energy of benzene molecule. “L3” and “L4” stand for neural networks with 3 and 4 layers, respectively. The CCSD(T) result coincides with our best DMC result with the 4-layer network. **e** The trend of node difference to final state in the training process together with the ones for VMC and DMC energy for a benzene molecule using a 3-layer FermiNet. **f** The linear trend between the node difference and the DMC energy difference to final state. The points correspond to different intermediate training steps and the dashed line is fitted using least square.

To further illustrate the power of our approach, we have examined the benzene molecule and a benzene dimer. Benzene is one of the most fundamental organic molecules with a hexagonal ring of C–H (Fig. 4a). There have been challenges in understanding its electronic configuration, bonding order and obtaining the ground state energy. To understand the electronic structure of benzene molecule, we performed FermiNet-based VMC and DMC simulations with 3-layer and 4-layer networks separately. Our best FermiNet-DMC result calculated with the 4-layer network coincides with the CCSD(T) result extrapolated to complete-basis-set (CBS) limit. The comparison is shown in Fig. 4d. The CCSD(T) result is carried out with Psi4^36^ and the CBS result is extrapolated using cc-pCVXZ (X=3,4,5) basis, which is much larger than the ones reported in Johnson III.^37^ and used by others as the state-of-the-art electronic structure methods in Eriksen et al.^38^. The energy from our CCSD(T)/CBS calculation is also much lower than those references. See Supplementary Note 14 for more details on the CCSD(T) calculation and CBS extrapolation.

The 3-layer FermiNet here is much smaller than the 4-layer one. Besides being one layer shallower, the number of neurons on each layer is also significantly less. See Supplementary Tables 8–10 for the related hyperparameters. Figure 4d shows that the 3-layer FermiNet-DMC’s energy is lower than the 4-layer VMC result by around 10 mHa, which demonstrates one of the main benefits of FermiNet-DMC that it can achieve better accuracy with smaller network. This is especially important when we are dealing with large systems.

In our calculations, FermiNet-DMC is able to achieve lower variational energy results with an order of magnitude better efficiency. With a total of 4 × 10^5^ QMC steps (2 × 10^5^ VMC training steps and 2 × 10^5^ DMC steps), the 3-layer FermiNet-DMC’s energy result (–232.225 Ha) is slightly better than the 4-layer FermiNet-VMC’s energy result (–232.223 Ha) at 10^6^ VMC training step. Moreover, the runtime of a single VMC step for the 4-layer network is approximately 4 times that of a single VMC or DMC step for the 3-layer network under the same computation resources. Therefore, in this case, the 3-layer FermiNet-DMC can achieve a better energy result at only a tenth of the total computation cost compared to the 4-layer FermiNet-VMC. Similarly, compared to the 3-layer FermiNet-VMC at 2 × 10^6^ VMC training step, the 3-layer FermiNet-DMC with 4 × 10^5^ QMC steps can achieve more than 10 mHa better energy result at only a fifth of the total computation cost.

Furthermore, the energy difference between the FermiNet-DMC results in Fig. 4d is only around 3 to 4 mHa, suggesting the closeness between the node structure of the two trial wavefunctions. To confirm this statement, we visualized 2-dimensional slices of those trial wavefunctions in Fig. 4b, c. The slices are generated by moving a single spin-up electron inside a two dimensional box while fixing all other electrons at representative positions suggested by Liu et al.^39^ and illustrated in Fig. 4a. See section “Nodal structure and wavefunction visualization” and Supplementary Note 11 for more visualization details. Comparing Fig. 4b (4-layer FermiNet VMC) and Fig. 4c (3-layer FermiNet), we find that the nodes, represented by the dark pixels, do share the same pattern. Moreover, the parts of nodal surface in lighter areas, namely with larger wavefunction value, are very close to each other in Fig. 4b, c, and they are the most important parts of nodal surface in the DMC process since walkers are more likely to visit its neighborhood. The closeness of those parts is consistent with the fact that the FermiNet-DMC energies are close.

To track how nodal surface evolves along the training process, we propose a divergence *D*(*S*, *T*) measuring the difference between two nodal surfaces *S* and *T*. The definition and algorithmic details are described in section “Divergence measuring nodal surface difference” and Supplementary Note 15, and the definition is also related to the intuition mentioned above that nodes in the neighborhood with larger wavefunction value are more important in the QMC calculation. For the 3-layer FermiNet, we calculated

$$D({S}_{{{{{\rm{final}}}}}},\,{S}_{k}),$$

where *S*_final_ and *S*_*k*_ are the nodal surface corresponding to the final VMC training step and the intermediate training steps *k*, respectively. The result is shown in Fig. 4e together with the VMC and DMC energy, where the trend of the divergence correlates well with energies. As a matter of fact, there is a linear relation between the divergence and DMC energy, as shown in Fig. 4f, indicating that the proposed divergence successfully captures the essential information of the difference between nodal surfaces. Here the divergence converges to around 0.005 instead of 0 because of the large learning rate used when training the 3-layer FermiNet for benzene.

**Fig. 5:**
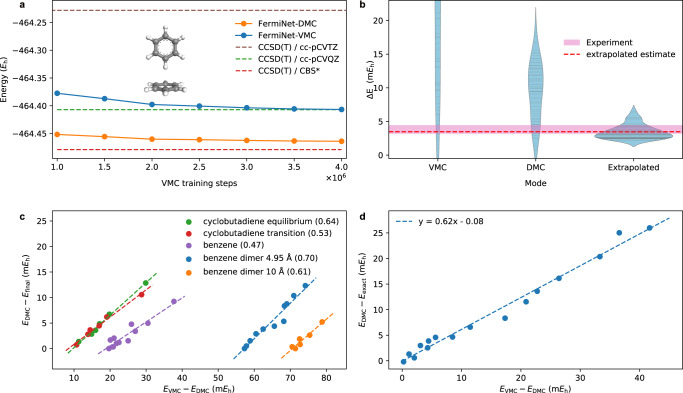
Benzene dimer calculated energy and the extrapolation based on VMC-DMC linear relation. **a** The energy of the T-shaped benzene dimer with a center-to-center distance of 4.95 Å as well as the CCSD(T) results as baselines. The CCSD(T)/CBS* result is calculated using binding energy and monomer energy. See Supplementary Note 14 for details. **b** The fitted distribution of binding energy from VMC, DMC, and extrapolation results, where the solid lines inside each violin represent the actual data points. The energy for two separated benzene molecules is calculated using a benzene dimer separated by 10 Å^43^. The equilibrium configuration is a T-shaped structure with center-to-center distance of 4.95 Å^40^. The experimental range is from Grover et al.^46^. The extrapolation scheme is based on the empirical linear relation between VMC energy (*E*_VMC_) and DMC energy (*E*_DMC_) in the training process, indicated by the blue and yellow points in **c**. **c** Linear fitting of *E*_VMC_ − *E*_DMC_ with respect to *E*_DMC_ − *E*_final_ on various molecular systems, where *E*_final_ represents the DMC energy at final VMC training step. The number within the parenthesis in the legend is the fitted slope for each system. **d** Linear fitting of *E*_VMC_ − *E*_DMC_ with respect to *E*_DMC_ − *E*_exact_ on atoms, where the energy data are the same as Fig. 2e. Note that the data in **d** is fundamentally different from **c** in the sense that the linearity in **c** is about different training steps for the same system while in **d** the linearity is measured across different systems.

We have also trained a neural network for a benzene dimer, which is a prototypical system to further test non-covalent interactions. Benzene dimer, which has 84 electrons in total, is a much larger system than the ones considered in previous neural network-based VMC works^19–26^. We elaborate the challenges and tricks dealing with large systems using FermiNet-based QMC methods in Supplementary Note 5. We consider a T-shaped structure with an edge-to-face arrangement, as illustrated in Fig. 5a, specifically the equilibrium configuration with a center-to-center distance of 4.95 Å^40^. Figure 5a also shows the VMC and DMC energies as functions of the VMC training step, which are both over 200 mHa lower than the CCSD(T) result with cc-pCVTZ basis. The converged FermiNet-DMC energy is over 50 mHa lower than both FermiNet-VMC result and the CCSD(T) result with cc-pCVQZ basis. It echos statements made in above sections that FermiNet-DMC can achieve significantly higher accuracy for larger systems or cases where the neural network ansatz is not powerful enough to characterize the ground state wavefunction well. For comparison, the FermiNet-VMC energy has not fully converged even after four million training steps. Schätzle et al.^41^ shows that neural network-based VMC, in particular, PauliNet, can achieve variational energies at the fixed-node limit in certain circumstances, while in our calculations, one can clearly see that it is not the case for FermiNet especially when its expressive power is limited compared to the size of the system. On the other hand, our DMC result is 15 mHa higher than the CCSD(T)/CBS result. Note that CCSD(T) is not a variational method, hence the relatively lower CCSD(T)/CBS result may indicate similar accuracy compared to our DMC result. To achieve more accurate FermiNet-DMC result, we can use a better neural network trial wavefunction with a larger network or a better network architecture.

In addition to the total energy at the equilibrium configuration, binding energy is also of great interest when studying a benzene dimer^40,42–45^, and classical methods, such as CCSD(T) and MP2, can produce results agreeing with experimental data well. However, for neural network-based QMC, the binding energy calculation is more subtle and challenging due to the lack of systematic error cancellation. Using the same network structure handling both monomer and dimer would introduce additional size-inconsistency-like bias because of the more severe expressiveness limitation on benzene dimer than monomer. For the benzene dimer, we find such an estimate would predict a severe underbinding with both VMC and DMC. Another way to estimate the binding energy is to take the difference between a separated dimer configuration (10 Å)^43^ and the equilibrium configuration, shown in Fig. 5b, which turns out to be systematically overbinding. With an empirical linear relation between VMC and DMC energy in the training process, we developed a simple VMC-DMC hybrid extrapolation scheme, which leads to an accurate estimate of the binding energy, well-agreed with the experimental measurements^46^, also displayed in Fig. 5b. We will elaborate more on this extrapolation scheme in section “Linear relation between VMC-DMC energy”. In order to systematically improve the binding energy calculation, the most straightforward way is to adopt better neural network ansatz as the trial wavefunction for better accuracy. Adding regularization mechanism in the optimization processes is another possible option so that the model variance can be reduced for better error cancellation. Note that in the case of DMC with pseudopotential, binding energy calculation can be also improved with certain deterministic approximation^47^. We will leave it as a future study apply those ideas to improve the binding energy calculation.

### Linear relation between VMC-DMC energy

Quite consistently, we find linear relation between VMC and DMC energies in our calculation. We have encountered two types of linear relation. One is about intermediate energies calculated along the training process for a given system, while another one is about the converged energies from different systems. We take advantage of the first type of linearity and develop a simple but effective extrapolation scheme accordingly.

We find that, for molecular systems, such as cyclobutadiene, benzene monomer and dimers, there’s a linear trend between the VMC and DMC energies calculated at different steps along the VMC training process. Equivalently, there’s a linear relation between quantities

$${E}_{{{{{{{{\rm{DMC}}}}}}}}}^{(k)}-{E}_{{{{{{{{\rm{final}}}}}}}}}\,\,{{\mbox{v.s.}}}\,\,{E}_{{{{{{{{\rm{VMC}}}}}}}}}^{(k)}-{E}_{{{{{{{{\rm{DMC}}}}}}}}}^{(k)}$$

where ${E}_{{{{{{{{\rm{VMC}}}}}}}}}^{(k)}$ and ${E}_{{{{{{{{\rm{DMC}}}}}}}}}^{(k)}$ represent the VMC and DMC energy calculated at VMC training step *k*, and *E*_final_ is the DMC energy at the final VMC training step, namely a constant for one training process. Such relation is shown in Fig. 5c. Based on this empirical linear relation, we propose an extrapolation scheme

1 $${E}_{{{{{{{{\rm{DMC}}}}}}}}}^{(k)}-{E}_{{{{{{{{\rm{ex}}}}}}}}}=w\cdot \left({E}_{{{{{{{{\rm{VMC}}}}}}}}}^{(k)}-{E}_{{{{{{{{\rm{DMC}}}}}}}}}^{(k)}\right)+b$$

where *E*_ex_ is the extrapolated energy, and *w* and *b* are two parameters to be determined. Here slope *w* can be fitted using ${E}_{{{{{{{{\rm{VMC}}}}}}}}}^{(k)}$ and ${E}_{{{{{{{{\rm{DMC}}}}}}}}}^{(k)}$ along the training process, but the intercept *b* cannot be inferred from those data. Therefore, it is difficult to use this scheme to extrapolate absolute energy unless we have extra information on intercept *b*. On the other hand, when calculating relative energy, we may simply assume the intercept *b* between different configurations are the same so that it can be canceled out in the calculation. Namely for relative energy, we have

2 $$\Delta {E}_{{{{{{{{\rm{ex}}}}}}}}}=(1+w)\cdot \Delta {E}_{{{{{{{{\rm{DMC}}}}}}}}}-w\cdot \Delta {E}_{{{{{{{{\rm{VMC}}}}}}}}}$$

Note that the calculation of relative energy is especially troublesome for neural network-based QMC methods, due to the strong dependence on the number of training steps and the long converging period. See Supplementary Fig. 8b for how binding energies calculated with FermiNet VMC and DMC change along the optimization process. With our scheme, the binding energy results calculated from different VMC training steps would be the same, modulo the fitting error of the linear relation, which means we can circumvent the dependence of the binding energy result on the number of training steps. In practice, the extrapolated binding energies form a well concentrated distribution, and doing an extra average using different VMC training steps can eliminate the linear fitting error and provide an accurate estimate. Moreover, it also suggests that we can calculate the extrapolated binding energy with data collected in the early phase of the training process, avoiding the long converging period of VMC optimization.

Applying this scheme to binding energy calculation of a benzene dimer, the result is significantly improved and the distribution fitted from energy difference of different VMC training steps is concentrated around the experimental range, as shown in Fig. 5b. The estimate of extrapolated binding energy by averaging the energy difference is 3.60 mHa, within the experimental range. See Supplementary Note 13 for more extrapolation-related details for benzene dimer.

We have discussed the relation of VMC and DMC energy for elements on the second and third rows in section “Single atoms”. For each atom, we have a reference energy data *E*_exact_ to be compared with converged VMC energy (*E*_VMC_) and DMC energy (*E*_DMC_). As shown in Fig. 2e, both *E*_DMC_ − *E*_exact_ and *E*_VMC_ − *E*_exact_ grow linearly as the atomic number increases, though the slope changes when switching from the second row elements to the third row. However, if we instead compare

$${E}_{{{{{{{{\rm{DMC}}}}}}}}}-{E}_{{{{{{{{\rm{exact}}}}}}}}}\,\,{{\mbox{v.s.}}}\,\,{E}_{{{{{{{{\rm{VMC}}}}}}}}}-{E}_{{{{{{{{\rm{DMC}}}}}}}}},$$

then we have a single linear relation across all elements on both second and third rows, as shown in Fig. 5d.

Interestingly, the slope of fitted lines in both Fig. 5c and d are all quite close. We will leave further study on those two types of linearity as future work.

## Discussion

FermiNet-DMC is able to achieve accurate ab initio calculations for various systems, obtaining ground state of 16 atoms, N_2_ along the bonding curve, 2 cyclobutadiene configurations, 10 hydrogen bonded water dimers, benzene monomer and dimer. These systems include bond breaking structures where strong static correlation exists and weakly bonded dimers where dynamic correlation dominates, and FermiNet-DMC performs consistently well. FermiNet-DMC leverages the expressive power of neural network to provide well-behaved trial wavefunctions. Neural network-based VMC has claimed success in small systems when the network can be sufficiently trained. However, it is not able to provide satisfactory ground state wavefunction and energy when the expressiveness of the neural network is limited. Compared to VMC, the combination of neural network with DMC provides a powerful solution, in the sense that it can achieve more accurate result with simpler network and better efficiency. The improvement of FermiNet-DMC in efficiency can be up to 1 or 2 orders of magnitude in the large systems tested in order to reach the same accuracy level as FermiNet-VMC, which can become increasingly more important when dealing with even larger molecules.

There is an interesting linear relation between VMC and DMC energy observed during the training process as well as across different systems. We develop an extrapolation scheme accordingly, which greatly improves the accuracy of relative energy calculation as shown in the benzene dimer case and overcome the issue that the relative energy calculation greatly depends on the different training steps in the QMC process. We also design a divergence measuring the difference between nodal surfaces of two wavefunctions, which correlates well with the corresponding DMC energies in numerical experiments. Namely the proposed divergence successfully captures the essence of nodal surface differences.

It is worth pointing out that a similar idea to this work was proposed in a preprint by Wilson et al., where they have performed preliminary tests on the second row elements^31^. However, only minor improvements in accuracy were observed accompanied by an increased cost of DMC, since the FermiNet used there was powerful enough to achieve high accuracy for the tested small systems and leave little room for further improvement. By comparison, our approach, being more sophisticated and efficient, achieves significant accuracy boost when dealing with more challenging molecular systems, which FermiNet alone cannot handle well. We have also shown that even for small systems, FermiNet-DMC should still be preferred for the fact that it can achieve comparable or even better accuracy with a smaller network and much less computation resources compared with FermiNet-VMC. Our work, therefore, eliminates the negative concerns of going from VMC to DMC with neural network wavefunction ansatz. Moreover, the DMC method can be further integrated with other powerful molecular neural networks^22,25^, periodic neural network for solids^48^, neural networks with effective core potential^49^, which has the potential to catalyze a paradigm shift in the application of stochastic electronic structure methods.

## Methods

### Basic theory

To study a many-body system from first principles, we always consider solving the well-known Schrödinger equation for electrons and nuclei. When we work in the Born-Oppenheimer approximation^50^, and further consider a fixed set of nuclear positions, the problem is simplified to the solution of the ground state many-electron wavefunction.

3 $$\hat{H}\psi ({{{{{{{{\bf{x}}}}}}}}}_{1},\cdots \,,{{{{{{{{\bf{x}}}}}}}}}_{n})=	 E\psi ({{{{{{{{\bf{x}}}}}}}}}_{1},\cdots \,,{{{{{{{{\bf{x}}}}}}}}}_{n}),\\ \hat{H}=	 -\frac{1}{2}\mathop{\sum}\limits_{i}{\nabla }_{i}^{2}-\mathop{\sum}\limits_{I}\mathop{\sum}\limits_{i}\frac{{Z}_{I}}{\left|{{{{{{{{\bf{r}}}}}}}}}_{i}-{{{{{{{{\bf{R}}}}}}}}}_{I}\right|}\\ 	+\mathop{\sum}\limits_{i < j}\frac{1}{\left|{{{{{{{{\bf{r}}}}}}}}}_{i}-{{{{{{{{\bf{r}}}}}}}}}_{j}\right|}+\mathop{\sum}\limits_{I < J}\frac{{Z}_{I}{Z}_{J}}{\left|{{{{{{{{\bf{R}}}}}}}}}_{I}-{{{{{{{{\bf{R}}}}}}}}}_{J}\right|},$$

where **x**_*i*_ = (**r**_*i*_, *σ*_*i*_) denotes the spatial and spin coordinates of electron *i*, and **R**_*I*_, *Z*_*I*_, respectively, denote the spatial coordinates and the charge of nucleus *I*. The wavefunction of electrons obeys Fermi-Dirac statistics thus should be antisymmetric with respect to the interchange of both the spatial coordinates and the spins of any two electrons, namely the following equality of wavefunction should hold: *ψ*( ⋯  , **x**_*i*_, ⋯  , **x**_*j*_, ⋯  ) = − *ψ*( ⋯  , **x**_*j*_, ⋯  , **x**_*i*_, ⋯  ).

Unlike most methods that use variational principle to approach the ground state wavefunction, DMC is a stochastic projection method. A given antisymmetric wavefunction *ψ*_*T*_ can always be represented as a linear combination of a set of eigenfunctions *ψ*_*k*_ of the corresponding Hamiltonian operator,

4 $${\psi }_{T}({{{{{{{{\bf{x}}}}}}}}}_{1},\cdots \,,{{{{{{{{\bf{x}}}}}}}}}_{n})=	\mathop{\sum }\limits_{k=0}^{\infty }{c}_{k}{\psi }_{k}({{{{{{{{\bf{x}}}}}}}}}_{1},\cdots \,,{{{{{{{{\bf{x}}}}}}}}}_{n}),\\ \hat{H}{\psi }_{k}({{{{{{{{\bf{x}}}}}}}}}_{1},\cdots \,,{{{{{{{{\bf{x}}}}}}}}}_{n})=	{E}_{k}{\psi }_{k}({{{{{{{{\bf{x}}}}}}}}}_{1},\cdots \,,{{{{{{{{\bf{x}}}}}}}}}_{n}),$$

When an imaginary-time evolution operator acts on *ψ*_*T*_,

5 $${e}^{-\tau (\hat{H}-{E}_{T})}{\psi }_{T}=\mathop{\sum }\limits_{k=0}^{\infty }{c}_{k}{e}^{-\tau ({E}_{k}-{E}_{T})}{\psi }_{k},$$

where *E*_*T*_ is the trial energy as an offset, there will be a decay coefficient added to each expansion term, and the decay rate is proportional to state energy *E*_*k*_. After a long enough imaginary-time evolution, *ψ*_*T*_ can reach the ground state *ψ*_0_, whereas contributions from all other eigenfuntions vanish. If we define a time-dependent wavefunction and look at the imaginary-time Schrödinger equation:

6 $$\psi ({{{{{{{{\bf{x}}}}}}}}}_{1},\cdots \,,{{{{{{{{\bf{x}}}}}}}}}_{n},\tau )=	{e}^{-\tau (\hat{H}-{E}_{T})}{\psi }_{T}({{{{{{{{\bf{x}}}}}}}}}_{1},\cdots \,,{{{{{{{{\bf{x}}}}}}}}}_{n}),\\ -{\partial }_{\tau }\psi ({{{{{{{{\bf{x}}}}}}}}}_{1},\cdots \,,{{{{{{{{\bf{x}}}}}}}}}_{n},\tau )=	(\hat{H}-{E}_{T})\psi ({{{{{{{{\bf{x}}}}}}}}}_{1},\cdots \,,{{{{{{{{\bf{x}}}}}}}}}_{n},\tau ).$$

Without the potential energy terms, it resembles a standard diffusion equation,

7 $${\partial }_{\tau }\psi ({{{{{{{{\bf{x}}}}}}}}}_{1},\cdots \,,{{{{{{{{\bf{x}}}}}}}}}_{n},\tau )=\frac{1}{2}\mathop{\sum}\limits_{i}{\nabla }_{i}^{2}\psi ({{{{{{{{\bf{x}}}}}}}}}_{1},\cdots \,,{{{{{{{{\bf{x}}}}}}}}}_{n},\tau ).$$

The diffusion equation defines the master equation of stochastic processes, hence we can solve the diffusion equation of wavefunction by simulating the stochastic processes^51^. With potential terms, additional processes are required to bind the diffusion equation in simulation (see, e.g., refs. ^16,52,53^ for more details).

### Trial wavefunction

In this work, we use FermiNet neural network ansatz as our trial wavefunction. Due to the huge number of parameters, it is challenging to converge the training process of FermiNet unless the system is small enough. After many tests, we identified a common training pattern of FermiNet, which consists of two stages: a relatively short sharp-adjustment stage and a lengthy fine-tuning one. We propose to use the FermiNet wavefunction right after the sharp-adjustment stage as the trial wavefunction in DMC, which maximizes the efficiency of the entire simulation protocol. In this way we can also achieve more accurate results than a better converged FermiNet model after the lengthy fine-tuning stage. Comparing to the gain, the cost of performing DMC on the long-trained FermiNet is rather minor in most of the systems tested.

### DMC implementation

We have developed a GPU-friendly DMC software in JAX^54^, which can be seamlessly integrated with FermiNet^27^, developed in the same programming framework. Our DMC software can also be integrated with other trial wavefunctions implemented in JAX and it has been open sourced in order to accelerate further combination of QMC methods with neural networks. See Algorithm 1 for a brief workflow of one DMC iteration, beyond which various of modifications are implemented, including those proposed by Umrigar et al. to reduce time-step error^52^ and by Zen et al. to keep size consistency^55^.

Random walkers’ branching and merging change the total number of walkers, which cause efficiency issue for JAX program and is also not friendly to distributed computing especially when load balancing is involved. We devised a new branching-merging strategy to overcome these issues. Whenever we need to branch certain random walker due to its overly large weight, we also merge two walkers on the same computing node with the smallest weight. No merging is executed if no branching happens. In this way, we keep the number of walkers on each computing node unchanged. We did thorough numerical verification of this strategy and found that the introduced bias is negligible.

The most time-consuming module in our DMC implementation is to calculate the local energy. In our optimized program, the computational cost for each local energy estimation is almost same as a VMC inference step of the original FermiNet. Therefore, the total cost depends solely on the number of iterations performed in DMC and VMC.

### Energy calculation

For FermiNet-VMC, we always perform a separate inference simulation for energy estimate, where we fix all the parameters of FermiNet after training and do a number of Markov Chain Monte Carlo (MCMC) steps to sample batches of random walkers accordingly. We calculate the average local energy for each batch, and use reblock analysis to determine the mean value of the set of averaged energy as well as the standard deviation. For FermiNet-DMC, we use the mixed estimator of energy^52^ and treat the first 10% of MC steps as the equilibrating phase and only use the steps afterwards for energy production. See Supplementary Tables 6–12 for the hyperparameters of all our calculations. We also use reblock analysis to determine the mean of the averaged energy and its standard error. In our plots, error bars represent one standard error for energy estimates, unless otherwise specified.

#### Algorithm 1

Simplified Diffusion Monte Carlo algorithm pseudocode. 

Note that walkers in DMC are more auto-correlated than the ones in VMC inference phase especially when the time-step used in DMC is set to be small to avoid bias. Therefore, more batches of random walkers are needed to reduce the statistical error to a given level in DMC than in VMC. However in practice, we found that the number of the required extra batches of walkers in DMC is usually much fewer than the number of steps in VMC training phase for full convergence.

### Nodal structure and wavefunction visualization

The three-dimensional cuts of the full 11D nodal structure of Be in Fig. 2b–d is plotted according to the rules of Bressanini et al.^28^. The four electrons’ spherical coordinates are respectively

$$\left\{\begin{array}{lll}{r}_{1}\in [0.1,2.1]\,{{{{{{{\rm{a.u.}}}}}}}},&{\phi }_{1}=0,\hfill &{\theta }_{1}\in [-1,\,1],\hfill \\ {r}_{2}=1.1\,{{{{{{{\rm{a.u.}}}}}}}},\hfill &{\phi }_{2}=0,\hfill &{\theta }_{2}=\pi /2,\hfill \\ {r}_{3}\in [0.1,2.1]\,{{{{{{{\rm{a.u.}}}}}}}},\hfill &{\phi }_{3}=\pi /2,\hfill &{\theta }_{3}=\pi /2,\hfill \\ {r}_{4}=1.1\,{{{{{{{\rm{a.u.}}}}}}}},\hfill &{\phi }_{4}=3\pi /2,&{\theta }_{4}=\pi /2,\hfill \end{array}\right.$$

fixing all the degrees of freedom except *r*_1_, *θ*_1_ and *r*_3_. The green surfaces in the plots show the nodal surfaces, i.e., the places where the value of wavefunction is zero.

To visualize the nodal surface of benzene, we calculated the wavefunction value on 2-dimensional slices of the 126-dimensional space. We first fixed a 126-dimensional electron configuration at the representative position of benzene electronic structure from Liu et al.^39^, and perturb it slightly for the visualization purpose. To construct one slice of the 126-dimensional space, we move a single spin-up electron in a 2-dimensional square with all other 41 electrons fixed. Then we apply FermiNet to points on each slice and display the log-scaled magnitude of the evaluated wavefunction value, where the points with small value stand for the nodes on each slice. Since the FermiNet output is unnormalized, diagrams for different FermiNet may have drastically different range of displayed value.

### Divergence measuring nodal surface difference

We define a divergence measuring the difference between two sets *S*_1_ and *S*_2_ in any metric space as follows

8 $$D({S}_{1},{S}_{2})={E}_{Y \sim {P}_{1}}d(Y,{S}_{2})\approx \mathop{\sum }\limits_{i}^{K}d({Y}_{i},{S}_{2})$$

where *P*_1_ is a probability measure on *S*_1_, and ${\{{Y}_{i}\}}_{i=1,\ldots,K}$ are sampled from *P*_1_. The distance *d*(*Y*, *S*) between a single point *Y* and a set *S* is defined as the smallest distance between *Y* and any point in *S*, namely

9 $$d(Y,S)=\mathop{\min }\limits_{Z\in S}d(Y,Z)$$

For a nodal surface S corresponding to an unnormalized wavefunction Ψ, we would like to define a measure on S such that a small area on S is assigned larger weight if its neighborhood has larger Ψ^2^ value, namely larger probability to be visited by walkers in DMC. Therefore, we consider a neighborhood

10 $${S}_{\epsilon }=\{x|d(x,\,S) \, < \, \epsilon \}$$

around S and a mapping

$$\phi :{S}_{\epsilon }\to S,$$

then “push forward" the probability density ${m}_{{\Psi }^{2}}$ (corresponding to Ψ^2^) from *S*_*ϵ*_ to S via *ϕ*, namely

11 $$\phi \circ {m}_{{\Psi }^{2}}(n):=\,{m}_{{\Psi }^{2}}({\phi }^{-1}(s))=\frac{{\int}_{{\phi }^{-1}(s)}{\Psi }^{2}}{{\int}_{{S}_{\epsilon }}{\Psi }^{2}},\quad \forall \,{{\mbox{set}}}\,\,s\subset S$$

Intuitively, for any point *y* in *S*_*ϵ*_ we may simply choose *ϕ*(*y*) to be the point on *N* that is closest to *y*.

However, it’s quite difficult to determine both *S*_*ϵ*_ and *ϕ* mentioned above algorithmically, and thus, in practice, we use some approximate alternatives that are much easier to compute. See Supplementary Note 15 for the algorithmic detail.

## Supplementary information

Supplementary Information

## Data Availability

All data supporting the findings of this study are provided in Supplementary Information.

## Code availability

We have released our DMC software at https://github.com/bytedance/jaqmc.
