# Supplementary material for: Towards the ground state of molecules via diffusion Monte Carlo on neural networks
Source: Nat Commun. 2023 Apr 3;14:1860. doi: 10.1038/s41467-023-37609-3 (PMC10070323; doi:10.1038/s41467-023-37609-3)
Supplement: Supplementary file 1 — Supplementary Information [file 41467_2023_37609_MOESM1_ESM.pdf]

Supplementary Information: Towards the ground state of molecules via diffusion Monte Carlo on neural networks

Weiluo Ren[1], Weizhong Fu[1,2], Xiaojie Wu[1], and Ji Chen[2,3]

[1]*ByteDance Research, Zhonghang Plaza, No. 43, North 3rd Ring West Road, Haidian District, Beijing, People's Republic of China*
[2]*School of Physics, Peking University, Beijing 100871, People's Republic of China*
[3]*Interdisciplinary Institute of Light-Element Quantum Materials, Frontiers Science Center for Nano-Optoelectronics, Peking University, Beijing 100871, People's Republic of China*

Supplementary Note 1. Additional software detail

In the method section, we have briefly described the DMC software developed for this work, including key methodological details. Here we add a few more points, and a complete description of our software will be published elsewhere.

Our implementation of DMC is scaling-out friendly. We have tested our software on a GPU cluster with 16 computing nodes and 128 Nvidia V100 GPU cards, and obtained near-linear speed-up. Our software saves checkpoint for DMC periodically and uploads to a remote storage cluster so that it can be resilient to occasional preemption due to cluster scheduling and device failure, and it is able to continue the process with the most recent checkpoint downloaded when a rescheduling is triggered.

In order to support multi-node computation efficiently, we have to minimize the inter-nodes communication, listed below:

- We calculate the total energy with local energy and weights for all the walkers across all the nodes.
- We also calculate several metrics with across-node aggregation, such as acceptance rate, number of outliers and etc.
- We synchronize the action of uploading and downloading checkpoints.

We have not yet supported across-node load balancing in terms of walkers, which could cause large communication overhead. As a matter of fact, we have devised a branching-merging algorithm which fixes the walker size per computing node so that we do not need to apply across-node walker load balancing at all. Every time when we do branching at the presence of a large-weight walker, we simultaneously find two walkers on the same node but with the smallest weight and merge them. Note that if the trial wavefunction has good quality and we have a decent number of walkers on each node, then such an action does not introduce much bias, confirmed in our calculations and shown in Supplementary Note 3. We only use the fixed-size branching scheme for relatively large systems, such as cyclobutadience, benzene molecule and benzene dimers, in which cases efficiency is crucial due to the computation power limit and the fixed-node error dominates the extra bias introduced by this branching scheme.

In order to revert the effect of changing trial energy, the energy produced from each DMC step should be corrected accordingly before averaged. However, as pointed in [1], when the batch size is large enough, the effect of such correction is negligible, which is consistent with our experience. Furthermore, it is easier to do reblocking analysis [2] to assess statistical error without such correction. Therefore, for all the results reported here, we do not include such energy corrections in our computation.

We have also implemented the electron-by-electron scheme suggested by CASINO [3], where each walker moves electrons one by one rather than all at once. Our tests show that the electron-by-electron scheme leads to a significant increase in the acceptance ratio of walkers with large time-steps, but we do not see much improvement on the time-step error or auto-correlation period. Besides, our electron-by-electron implementation sequentially goes over all the electrons in one configuration, leading to a decline in computing efficiency when dealing with large systems. So this option is also switched off by default.

Supplementary Note 2. Finite time-step error

We have implemented the ZSGMA approach proposed by Zen et al. to reduce the finite time-step error [4]. As tested in various systems in literature our default setting 10^{-3} is usually small enough to reach convergence. Here we show a test on the cyclobutadience with equilibrium structure as an example. The results are displayed in Supplementary Fig. 1, from which we can conclude that the finite time-step error for our default setting 10^{-3} is well below 1 mHa, comparable to the statistical error. Based on this observation as well as the efficiency concern, we use time-step 10^{-3} by default and use it in all of our FermiNet-DMC calculations.

To obtain comparable statistical error of the reported energy, we use different number of DMC steps for each time-step to compensate different level of auto-correlation in the energy time series. For time-step 10^{-3}, we run DMC for 10^5 steps. For large time-steps like 0.01 or 0.03, we only run DMC for 10^4 steps while for small time-step like 10^{-4}, we run DMC for 10^6 steps.

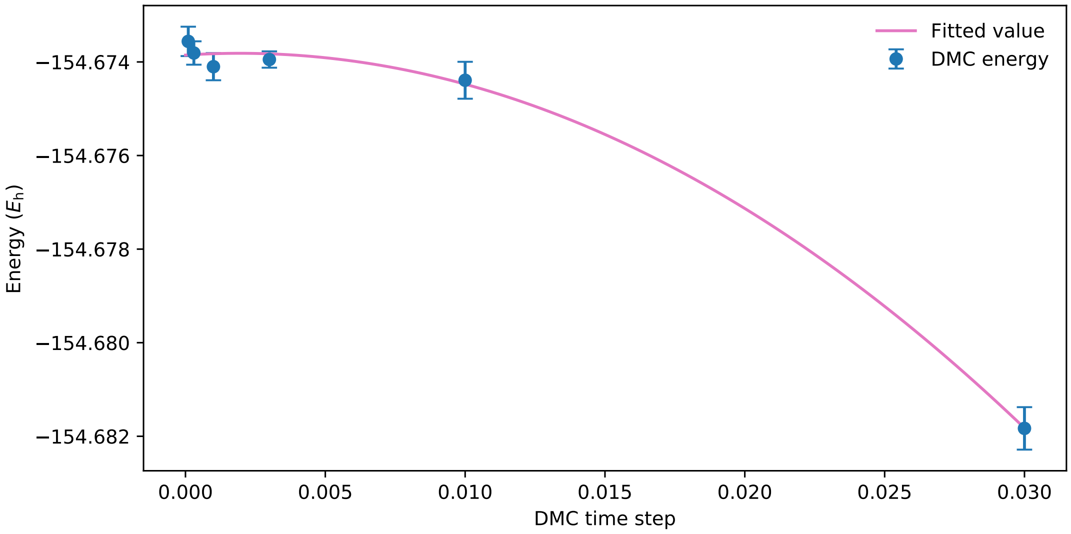

Supplementary Figure 1 | Time-step dependence of the total energy of cyclobutadience with equilibrium structure. The pink curve is generated by doing a quadratic fitting on the collected DMC energy data points.

Supplementary Note 3. DMC options

To investigate the effect of different hyperparameters of our DMC software, especially the bias introduced in different settings, we compare the DMC energy calculated with the settings listed in Supplementary Table 1, using cyclobutadience with equilibrium structure as an example. The trial wavefunction is a FermiNet trained with 10^5 steps. See Note 9 for more training details. The result is displayed in Supplementary Fig. 2. Taking the statistical error into account, all settings agree with each other well except the most inaccurate one with time-step 10^{-2}.

Supplementary Table 1 | We test our DMC software with different hyperparameters, listed as follows. “time step” is the DMC time-step. “equilibrium phase length” is the length of phase in the beginning of the whole DMC process during which we do not collect statistics for report purpose. “total number of steps” is the number of steps in the whole DMC process. “energy window size” is the length of the rolling window for trial-energy update. “fix size” indicates whether we fix the number of DMC walkers on each computing node or not. “energy cutoff” is the parameter α in the cutoff scheme proposed in [4] and indicates the level of energy clipping.

Identifier	time step	equilibrium phase length	total number of steps	energy window size	fix size	energy cutoff
time step 10^{-2}	10^{-2}	2×10^3	10^4	10^3	True	2
time step 10^{-3} (default)	10^{-3}	2×10^4	10^5	10^4	True	2
time step 10^{-4}	10^{-4}	2×10^5	10^6	10^5	True	2
window 5×10^4	10^{-3}	10^5	2×10^5	5×10^4	True	2
vary size	10^{-3}	2×10^4	10^5	10^4	False	2
energy cutoff 4	10^{-3}	2×10^4	10^5	10^4	True	4

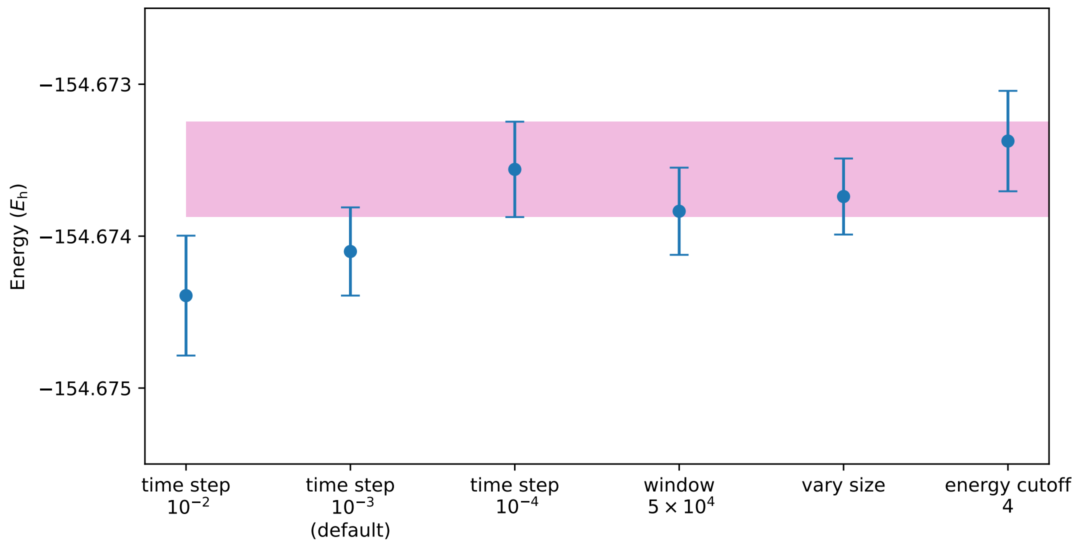

Supplementary Figure 2 | DMC energy and statistical error for different settings listed in Supplementary Table 1. The pink patch indicates the one-standard-error interval of energy in the most accurate setting with time step 10^{-4}. All the error bars are overlapped with this interval except the most inaccurate setting with time-step 10^{-2}.

Supplementary Note 4. Efficiency

In this section, we compare the efficiency between FermiNet-VMC [5–7] and FermiNet-DMC. The evaluation of FermiNet's local energy dominates the whole FermiNet-DMC process in terms of computation time. In other words, for one step, the difference between runtime of FermiNet-DMC and the inference phase of FermiNet-VMC is negligible.

However, DMC usually requires more steps to reduce the statistical error to a given level compared to VMC inference phase, because DMC has to use small time-step to avoid finite time-step error while VMC can use large time-step in Markov chain Monte Carlo (MCMC) to obtain less correlated samples. To cut down the statistical error more effectively in DMC, we can increase the batch size so that each batch contains more independent walkers. In practice, we start FermiNet-DMC procedure from the FermiNet-VMC walkers. To increase the DMC batch size, we duplicate the VMC walkers and then do MCMC for a period of time to get a larger batch of uncorrelated walkers, used as the starting walkers in DMC. Even if we do not enlarge the batch size, we still do a number of MCMC steps using fixed FermiNet before DMC so that the walkers is distributed closer to the square of FermiNet's wavefunction, which serve better starting point for DMC. Note that doing MCMC with FermiNet is much faster than doing DMC since no local energy calculation is involved. In our experiments, we use larger DMC batch size only when handling benzene dimers, in which case DMC uses batch size 16384 and VMC uses batch size 4096.

Overall FermiNet-DMC is still a much more efficient solution than FermiNet-VMC, because it can drastically cut down the length of the training phase of VMC which is significantly longer than the DMC itself. As a matter of fact, in the calculations that we performed, FermiNet-DMC can reach the same or even better accuracy level with a FermiNet trained with only one tenth or even a smaller fraction of total number of steps compare to the fully converged FermiNet-VMC.

Supplementary Note 5. Details on large systems

When dealing with relatively large systems containing dozens if not hundreds of electrons, chances are that we can not afford a large enough network to well represent the ground state wavefunction. Several issues emerge in this regime when training FermiNet. Firstly, the under-fit FermiNet may hit outliers hurting the convergence process. What's worse, the training process may hit NAN issue occasionally. We removed the outliers in terms of local energy when calculating the gradient in the FermiNet's training process. This helps to stabilize the calculation of the average and standard deviation of local energies in a batch, which benefits the gradient clipping process. Without such outlier-removal operation, at the presence of one or more outliers, the gradient clipping is not effective since we are not able to determine the clipping level correctly.

Secondly, the determinant calculation of large-dimension matrix is numerically troublesome. Potentially, there could be overflow and underflow issues especially when using single precision. Besides that, there is usually a dominating determinant among multiple determinants, which makes the value of other determinants effectively zero due to float number rounding. Therefore, we only use a single determinant when dealing with large systems so that the process becomes more efficient and stable. Admittedly, this limits the network expressiveness, and further improvements are left for future work.

Thirdly, the under-fitted decaying behavior of electron density at a distance may cause serious issues, introducing outliers in the calculation of total energy. For instance, there could be walkers occasionally diffusing away from atoms and reaching areas where the

neural network is not well trained, which causes trouble for the calculation of total energy and its gradient. We use isotropic envelope [6] to maintain the exponentially decaying behavior of the wavefunction when the electron is away from all the atoms, which also helps to improve the efficiency of both FermiNet-VMC and FermiNet-DMC.

Supplementary Note 6. Common Hyperparameters

All of our calculations follow the same procedure. First we train FermiNet with VMC[5–7], then we use the trained FermiNet as trial wavefunction in DMC. To obtain reliable energy from VMC, we run a separate inference process with the trained FermiNet. There are some hyperparameters shared by all the calculations in this work, as listed in Supplementary Table 2.

Supplementary Table 2 | Common hyperparameters

Hyperparameter	Value	Hyperparameter	Value
VMC optimizer	KFAC [8, 9]	Batch size	4096
Precision	Float32	Full determinant	True
DMC time-step	10^{-3}		

Supplementary Note 7. Atoms

A rather small neural network is used in calculations of single atoms (see Fig. 1e of main text), and all the training processes have well converged. The corresponding hyperparameters are listed in Supplementary Table 3.

Supplementary Table 3 | Hyperparameters for single atoms calculations in Fig. 2e.

Hyperparameter	Value	Hyperparameter	Value
Dimension of one electron layer	32	Dimension of two electron layer	4
Number of layers	2	Number of determinants	1
Envelope type	full	VMC learning rate	5×10^{-4}
Number of training steps	$\geq 5 \times 10^5$	Number of inference steps	10^5
Number of DMC steps	2×10^5	MCMC steps between each iterations	100
Outlier removal	false	Fixed-size branching	false

Supplementary Note 8. Nitrogen Molecule

Note that our FermiNet-VMC result shown in Fig. 3a of main text is better than the reported FermiNet result in [5]. The main difference is that our calculation uses the full determinant mode, which boosts the accuracy of FermiNet-VMC, as pointed out in [6, 10].

Supplementary Table 4 | Hyperparameters for nitrogen dissuciation curve calculations in Fig. 3a of main text.

Hyperparameter	Value	Hyperparameter	Value
Dimension of one electron layer	256	Dimension of two electron layer	32
Number of layers	4	Number of determinants	16
Envelope type	full	VMC learning rate	10^{-4}
Number of training steps	2×10^5	Number of inference steps	10^5
Number of DMC steps	2×10^5	MCMC steps between each iterations	10
Outlier removal	false	Fixed-size branching	false

Supplementary Note 9. Cyclobutadiene

We use the default network structure in the open-source FermiNet repository for cyclobutadiene, and train two neural networks for the equilibrium and transition state configurations separately. The training process was converged after 5×10^5 steps, as shown in Supplementary Fig. 3. We use FermiNet trained with 10^5 and 5×10^5 steps, and run FermiNet-DMC and FermiNet-VMC inference processes accordingly. The results are listed in Supplementary Table 6, where we also include the results from [6] for comparison.

Supplementary Table 5 | Hyperparameters for cyclobutadiene related calculations

Hyperparameter	Value	Hyperparameter	Value
Dimension of one electron layer	256	Dimension of two electron layer	32
Number of layers	4	Number of determinants	16
Envelope type	full	VMC learning rate	10^{-4}
Number of training steps	5×10^5	Number of inference steps	10^5
Number of DMC steps	10^5	MCMC steps between each iterations	10
Outlier removal	false	Fixed-size branching	true

Supplementary Table 6 | Cyclobutadiene ground state energy in Hartree. "VMC" and "DMC" indicates our FermiNet-VMC and FermiNet-DMC results respectively. For FermiNet-DMC, we include results corresponding to FermiNet trained with 10^5 and 5×10^5 steps. "VMC*" indicates results from [6].

Configuration	VMC		DMC		VMC*
	10^5 step	5×10^5 step	10^5 step	5×10^5 step	
Equilibrium	-154.6577(1)	-154.6655(1)	-154.6740(3)	-154.6770(2)	-154.6719(1)
Transition	-154.6430(1)	-154.6505(1)	-154.6585(3)	-154.6611(2)	-154.6554(1)

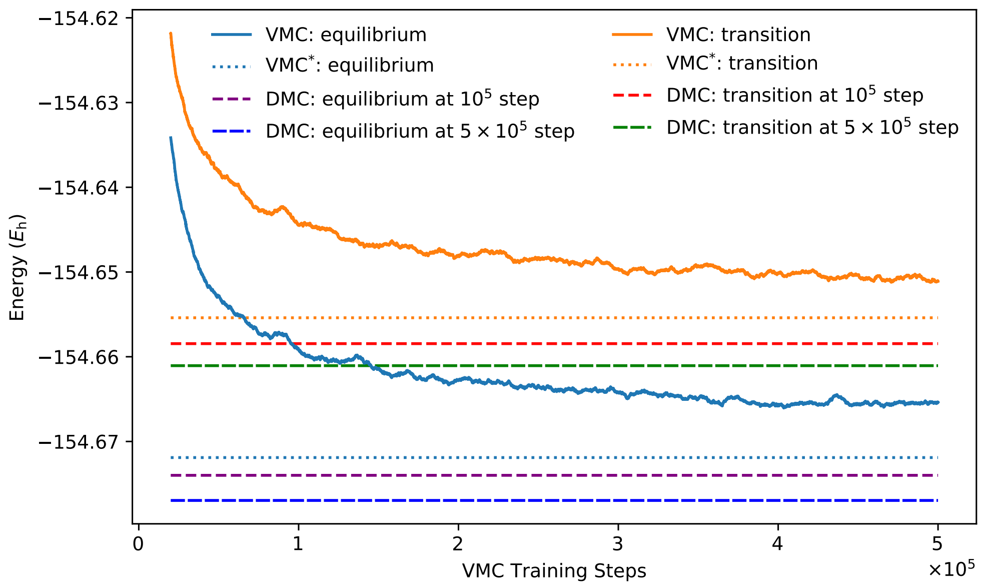

Supplementary Figure 3 | The FermiNet-VMC training curve and FermiNet-DMC energy for cyclobutadience equilibrium and transition configurations. "VMC" and "DMC" indicates our FermiNet-VMC and FermiNet-DMC results respectively. For FermiNet-DMC, we show results corresponding to FermiNet trained with 10^5 and 5×10^5 steps. "VMC*" indicates results from [6].

Supplementary Note 10. Water Dimer

The 10 structures of water dimer and their point-group symmetries are shown in Supplementary Fig. 4. The energy curves of FermiNet-VMC and corresponding FermiNet-DMC energy are shown in Supplementary Fig. 5, where all the VMC training curves show a clear "elbow" pattern and transition to the slow-converging phase around 10^5 step. The FermiNet-DMC energy at 10^5 step is already lower than the corresponding FermiNet-VMC energy at 3×10^5 step for all structures. Actually, for most structures the VMC energy curve is still not converged at 3×10^5 step. In principle, convergence in VMC training stage is necessary to investigate the energy difference. All the relative energy results are shown here in Supplementary Fig. 6. In Fig. 3d of main text we see that the mean absolute deviation of the relative energy results of FermiNet-DMC are much better than VMC's, especially at 10^5 step when the networks are undertrained. This is reasonable as the nodal surface, which contains the zero order information of wave function, naturally requires fewer training steps to converge. In conclusion, to calculate the relative energy, DMC is a much more efficient and reliable choice compared with VMC.

Supplementary Table 7 | Hyperparameters for water dimer calculations in Fig. 3d of the main text.

Hyperparameter	Value	Hyperparameter	Value
Dimension of one electron layer	256	Dimension of two electron layer	32
Number of layers	4	Number of determinants	16
Envelope type	full	VMC learning rate	10^{-4}
Number of training steps	3×10^5	Number of inference steps	10^5
Number of DMC steps	2×10^5	MCMC steps between each iterations	100
Outlier removal	false	Fixed-size branching	false

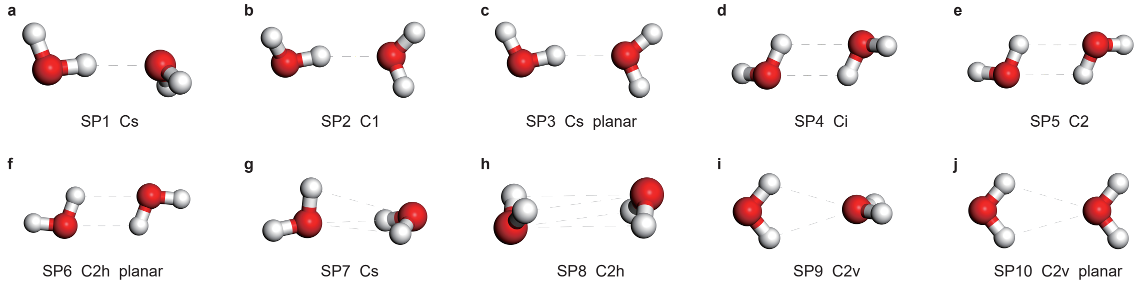

Supplementary Figure 4 | Geometries of the SPn ($n = 1, 2, ..., 10$) structures of water dimer, with their point-group symmetry labels.

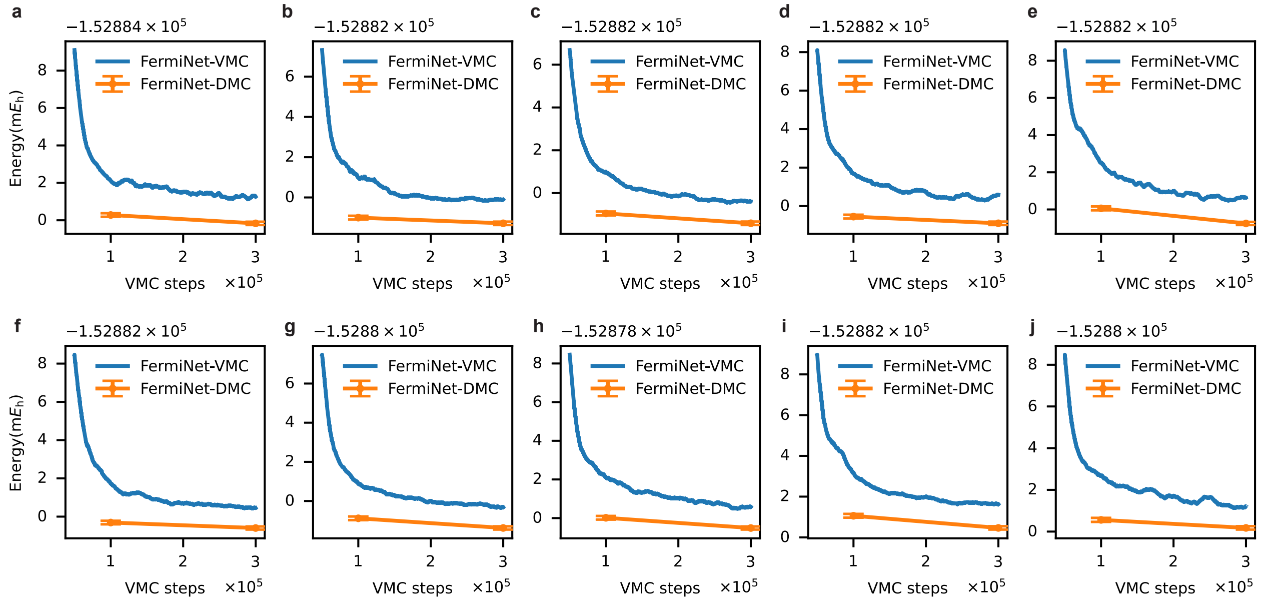

Supplementary Figure 5 | Total energy results of the SPn ($n = 1, 2, ..., 10$) structures of water dimer.

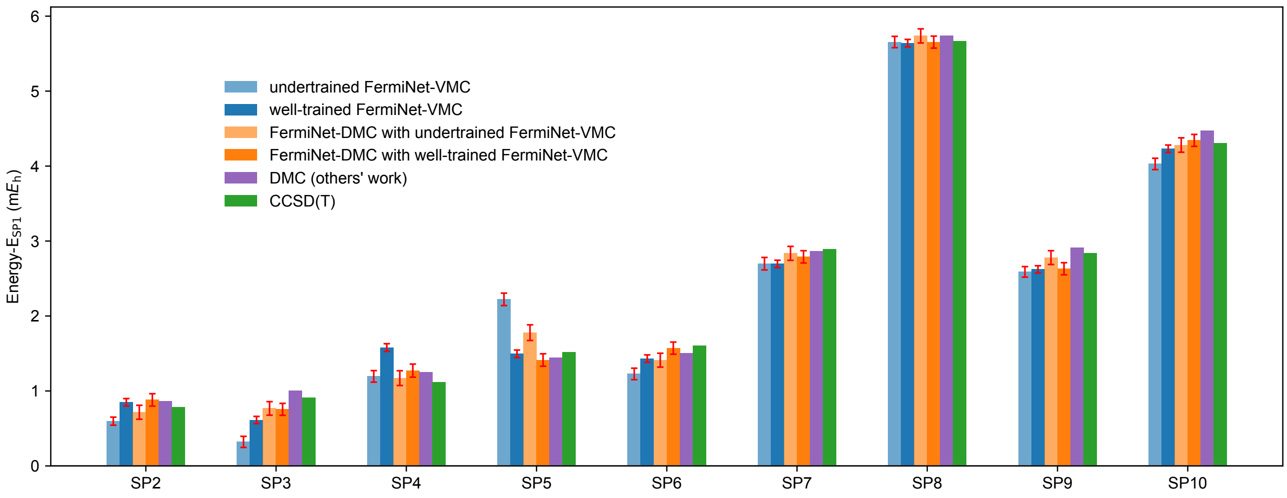

Supplementary Figure 6 | Relative energy results of the SPn ($n = 2, 3, ..., 10$) structures of water dimer to the SP1 structure.

Supplementary Note 11. Benzene

We trained a 3-layer and a 4-layer FermiNet for the benzene molecule. The common hyperparameters for both networks are listed in Supplementary Table 8. The hyperparameters indicating the network quality are described in Supplementary Table 9 and 10, respectively. The energy calculated are listed in Supplementary Table 11.

Supplementary Table 8 | Common hyperparameters for benzene related calculations

Hyperparameter	Value	Hyperparameter	Value
Number of determinants	1	Envelope type	isotropic
Number of VMC inference steps	10^5	Number of DMC steps	2×10^5
Learning rate	0.1	MCMC steps between each iterations	100
Outlier removal	True	Fixed-size branching	true

Supplementary Table 9 | Hyperparameters for 3-layer FermiNet for the benzene molecule

Hyperparameter	Value	Hyperparameter	Value
Dimension of one electron layer	128	Dimension of two electron layer	8
Number of layers	3	Number of VMC training steps	2×10^6

In Supplementary Fig. 7, we provide more visualization of the log scaled magnitude of wavefunction on slices of the electronic configuration space as supplement to the main text Fig. 4. As described in main text Section II F, the slices are generated by moving a single spin-up electron while keeping all others fixed in their representative positions. In Supplementary Fig. 7 , the moving electrons are corresponding to the C-H bonds in a benzene. Note that there is no symmetry built in the neural network, while we can still see clear patterns across different subplots in Supplementary Fig. 7, indicating that FermiNet successfully learned the symmetric property of this system. Moreover, the node structure near the light area, namely the one with larger wavefunction magnitude and larger probability to be sampled in the VMC process, shares higher level of similarity. Intuitively, this part of nodes plays more important roles in our fixed-node DMC compared to the rest of nodes which are less likely to be visited in the DMC process, further explaining why the 3-layer and 4-layer FermiNet-DMC results are so close.

Supplementary Table 10 | Hyperparameters for 4-layer FermiNet for the benenze moledule

Hyperparameter	Value	Hyperparameter	Value
Dimension of one electron layer	256	Dimension of two electron layer	32
Number of layers	4	Number of VMC training steps	10^6

Supplementary Table 11 | Calculated benzene energy with FermiNet based VMC and DMC in Hartree. The 3-layer neural network is trained with 2×10^6 steps and the 4-layer one is trained with 10^6 steps. The DMC energy has well converged.

Network Structure	FermiNet-VMC	FermiNet-DMC
3-layer	-232.2143(1)	-232.2330(3)
4-layer	-232.2233(1)	-232.2370(3)

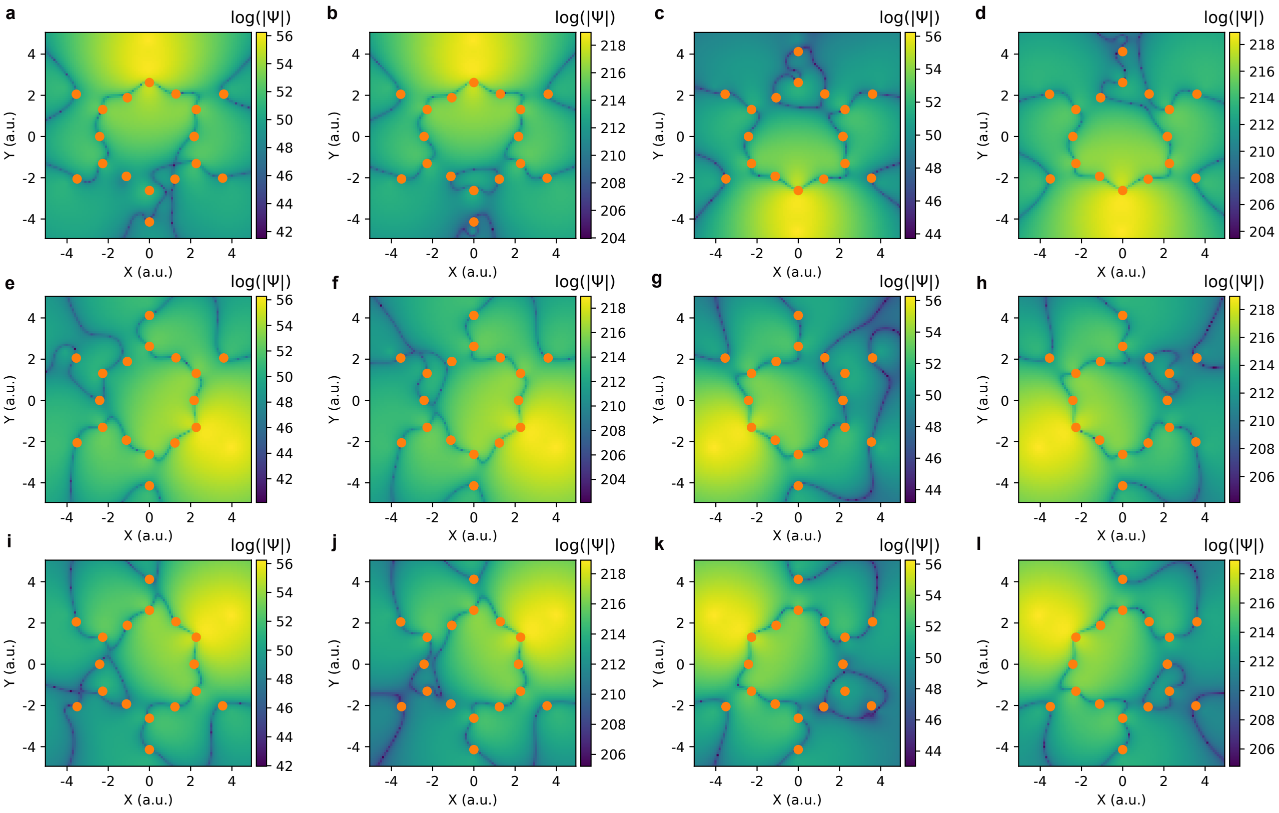

Supplementary Figure 7 | The 2-dimensional slices of log scaled magnitude of wavefunctions for benzene. The first and third column of figures are for a 4-layer FermiNet and the rest is for a 3-layer FermiNet. Each figure corresponds to a spin-up electron on a C-H bond, whose position is indicated by the lightest area in the figure. The dark pixels correspond to nodes.

Supplementary Note 12. Benzene dimer

We use the same 3-layer neural network for the benzene molecule to handle benzene dimers. The network for a benzene dimer is significantly more difficult to train than the benzene molecule. One of the tricky hyperparameters is the learning rate. Small learning rates lead to extremely slow convergence as well as trapping in local minimum, which was also mentioned in Lin et al. [10], while large learning rates may cause instability or even NaN issues ruining the whole process. In our numerous trials, we found that an initial learning rate of 0.01 with a slightly larger decay rate (5000 as opposed to the default 10000) hits a nice balance. It took millions of training steps to finally obtain a converge energy in FermiNet-DMC. In comparison, FermiNet-VMC has not converged even after four million steps. Our calculated energy for a T-shaped benzene dimer with bond length 4.95 Å is listed in Supplementary Table 12. The DMC energy of dimers are still higher than the doubled monomer DMC energy by 2 mHa, indicating that a 3-layer network is too restricted to handle a benzene dimer, which would lead to a bias when estimating binding energies.

Supplementary Table 12 | The ground state energy in Hartree for a T-shaped benzene dimer with bond length 4.95 Å. VMC and DMC results are based on a FermiNet trained for 4×10^6 steps.

Methods	Energy (E_h)
FermiNet-VMC	-464.4067(3)
FermiNet-DMC	–464.4640(2)

In principle, the binding energy can be accurately determined when the ground state energy of the dimer is calculated using a sufficiently large network, which however is beyond the capability of our available computational resource. In order to reduce the error in estimating the binding energy, we train a separate FermiNet for a configuration with two benzene molecules separated at 10 Å, which we dub as "long-distance". The training curve is also displayed in Supplementary Fig. 8.a. The calculated binding energy results, taken as the energy difference between the long-distance configuration and the equilibrium configuration, are shown in Fig. 8b. Although FermiNet-DMC improves the convergence and the final results of FermiNet-VMC significantly, it still shows a slight overbinding compared with experimental measured range. From the energy convergence pattern, the DMC energy for the long-distance configuration converges earlier than the one for the equilibrium configuration, resulting in an overly-large binding energy as the energy for equilibrium configuration gets smaller. The reason still lies in the inconsistency of two neural networks in describing two different configurations. To begin with, a long-distance configuration may be more difficult for FermiNet to handle, especially when its expressive power is limited. Similar phenomenon also shows up in [11] where DMC energy is too high for the long-distance configuration due to the limitation of the trial wavefunction. Moreover, the neural networks for different configurations are trained separately and therefore they may converge to local minima of different quality introducing additional bias in the binding energy calculation.

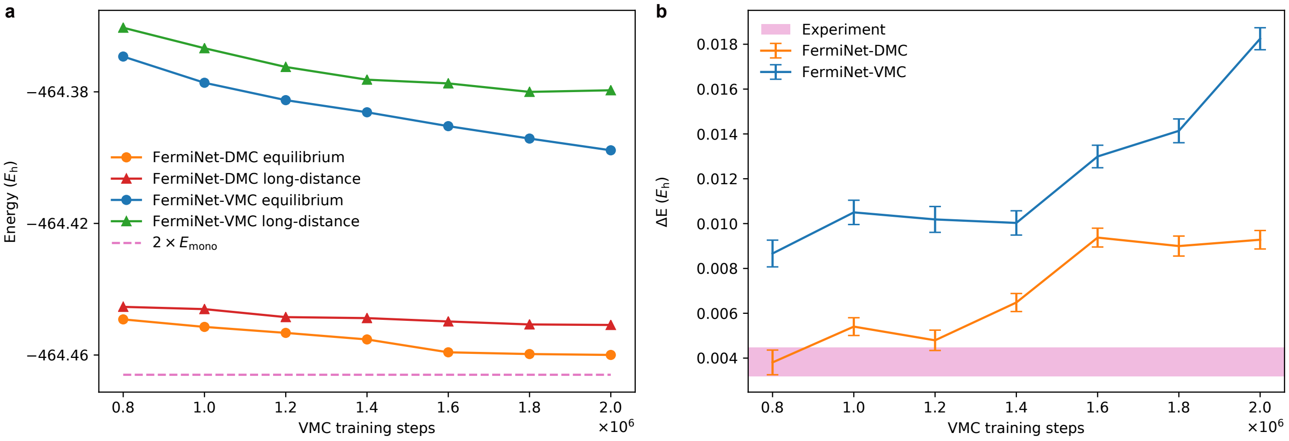

Supplementary Figure 8 | a. The energy curve for both equilibrium configurations with bond length 4.95 Å and long-distance configurations with bond length 10 Å calculated with FermiNet-VMC and FermiNet-DMC. **b**. Benzene dimer binding energy calculated with both FermiNet-VMC and FermiNet-DMC. Both results exceed the experimental range while FermiNet-DMC result is closer to the experimental upper limit.

Supplementary Note 13. Binding energy extrapolation

In this note we provide details on the binding energy extrapolation for benzene dimer discussed in main text Section II G.

We first show how to fit the slope parameter w in main text Eq. 1. For a given VMC training process, we do VMC inference and DMC process at a sequence of steps $\{k\}$ and collect VMC and DMC energy estimate $\{E^{(k)}_{\text{VMC}}\}$ and $\{E^{(k)}_{\text{DMC}}\}$. Following main text Eq. (1), we assume

$$E^{(k)}_{\text{DMC}} - E_{\text{ex}} = w \cdot (E^{(k)}_{\text{VMC}} - E^{(k)}_{\text{DMC}}) + b + \epsilon^{(k)} \tag{1}$$

where $\epsilon^{(k)}$ corresponds to 0-centered random noise.

A simple manipulation of Supplementary Eq. (1) leads to

$$E^{(i)}_{\text{DMC}} - E^{(j)}_{\text{DMC}} = w \cdot [(E^{(i)}_{\text{VMC}} - E^{(j)}_{\text{VMC}}) - (E^{(i)}_{\text{DMC}} - E^{(j)}_{\text{DMC}})] + (\epsilon^{(i)} - \epsilon^{(j)}) \quad \forall i, j \tag{2}$$

which can be used to fit for slope w with data $\{E^{(k)}_{\text{VMC}}\}$ and $\{E^{(k)}_{\text{DMC}}\}$ using least squares.

From main text Fig. 5c, we can tell that the slope of fitted line for benzene dimers with different bond length are very close. Therefore we choose to fit a single slope w with the data from two configurations combined, as shown in Supplementary Fig. 9.

With the fitted slope w_0, we have extrapolation from Supplementary Eq. (1)

$$E_{\text{ex, s}} = (1 + w_0) \cdot E^{(i)}_{\text{DMC, s}} - w_0 \cdot E^{(i)}_{\text{VMC, s}} - b - \epsilon^{(i)}_s$$

where s denotes the bond length for the benzene dimer and can choose value from 4.95 and 10 in this case. Therefore,

$$\Delta E_{ex} = E_{\text{ex, 10}} - E_{\text{ex, 4.95}} = (1 + w_0) \cdot (E^{(j)}_{\text{DMC, 10}} - E^{(i)}_{\text{DMC, 4.95}}) - w_0 \cdot (E^{(j)}_{\text{VMC, 10}} - E^{(i)}_{\text{VMC, 4.95}}) - (\epsilon^{(j)}_{10} - \epsilon^{(i)}_{4.95}) \tag{3}$$

Namely, with this scheme, we can get the extrapolated binding energy ΔE_{ex} from energy difference from any pair of training steps, modulo the fitting error. And this explains why the distribution of our extrapolated result concentrates so well in main text Fig. 5b. By averaging the Supplementary Eq. (3) over all chosen steps i and j, we significantly reduce the fitting error and thus get a much more accurate binding energy estimate, also shown in main text Fig. 5b.

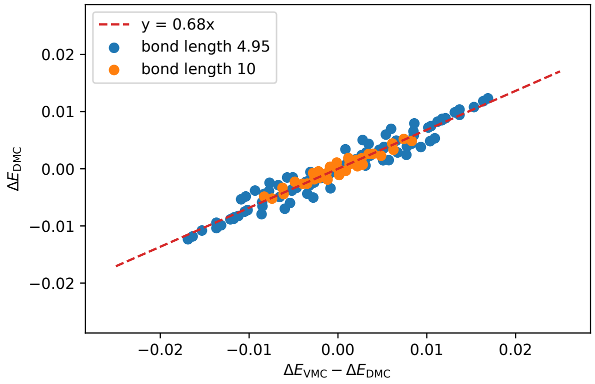

Supplementary Figure 9 | . For a pair of steps i and j in VMC training for benzene dimer, we plot two quantities showing up in Supplementary Eq. (2), namely $[(E^{(i)}_{\mathrm{VMC}} - E^{(j)}_{\mathrm{VMC}}) - (E^{(i)}_{\mathrm{DMC}} - E^{(j)}_{\mathrm{DMC}})]$ v.s. $E^{(i)}_{\mathrm{DMC}} - E^{(j)}_{\mathrm{DMC}}$. The data for benzene dimer with bond length 4.95 Å and 10 Å have very close slope. With those two set of data combined, we fit a straight line with least squares.

Supplementary Note 14. CCSD(T) calculations and their extrapolations

All CCSD(T) results in this paper are obtained with Psi4 [12]. Four-center electron repulsions are approximated with density fitting. Instead of the usual Dunning basis sets (cc-pVXZ) for valence-only calculation, we used Dunning basis sets (cc-pCVXZ) for valence-core correlations which approximate the exact solution to the all-electron problem. Since the corresponded auxiliary basis set for cc-pCV5Z is not available in the current version of Psi4 (v1.6), we assigned cc-pV5Z-jkfit as the auxiliary basis for cc-pCV5Z basis.

For the extrapolation of complete basis set (CBS) limit, we follow the exponential form [13]

$$E(X) = E_\infty + Be^{-\alpha X} \tag{4}$$

where X is the cardinal number corresponding to the number of basis functions for each atomic orbital (e.g. 2 for cc-pCVDZ, 3 for cc-pCVTZ). E_∞ is the energy at CBS limit. B and α are parameters needed to fit. Hartree-Fock energy, CCSD correlation energy, and (T) contribution are fitted separately using the same formula. Then three extrapolated contributions are added to the CCSD(T)/CBS result. We denote CBS(2-4) and CBS(3-5) as the extrapolations using cc-pCVXZ (X=2,3,4), and cc-pCVXZ (X=3,4,5) respectively. The detailed calculation for benzene is summarized in Table 13.

Supplementary Table 13 | Extrapolation of CBS limit for benzene molecule

basis	HF	CCSD	CCSD(T)	E_{corr}	(T) contribution
cc-pCVDZ	-230.724368	-231.777896	-231.815816	-1.053528	-0.037920
cc-pCVTZ	-230.779735	-232.057476	-232.112446	-1.277741	-0.054970
cc-pCVQZ	-230.793378	-232.143423	-232.202980	-1.350045	-0.059557
cc-pCV5Z	-230.796283	-232.167077	-232.227835	-1.370788	-0.060758
CBS(2-4)	-230.797839		-232.243544	-1.384460	-0.061245
CBS(3-5)	-230.797069		-232.237386	-1.379133	-0.061184

Our best estimate of CCSD(T)/CBS for benzene is −232.237386 Ha, given by CBS(3-5). The difference between CCSD(T)/CBS(2-4) and CCSD(T)/CBS(3-5) is about 6 mHa. We expect the extrapolation error of CBS(3-5) for Benzene molecule to be of the order of 1 mHa. The uncertainty of the extrapolation scheme is mostly contributed by the extrapolation of E_{corr}. Hartree-Fock energy and (T) contribution converge fast with respect to the basis set. Their uncertainties (difference between CBS(2-4) and CBS(3-5)) are less than 1 mHa, and almost negligible.

For Benzene dimer, the largest basis set we calculated is cc-pCVQZ. Hence, our best result for Benzene dimer is obtained by CBS(2-4), which gives −464.486530 Ha. We expect the extrapolation error of CBS(2-4) for Benzene dimer is of the order of 10 mHa, due to the uncertainty of CBS(2-4) and the doubled system size compared to a single Benzene molecule. We also calculated CCSD(T)/CBS(2-4) binding energy $E_{\mathrm{binding}} = 2 \times E_{\mathrm{mono}} - E_{4.95}$ where E_{mono} is the energy of benzene molecule, and $E_{4.95}$ is the energy of benzene dimer at 4.95 Å. The binding energy is calculated for each finite basis set first. Then, they are extrapolated with Supplementary Eq. (4). The binding energy converges with respect to the basis set much faster than the absolute energy does (Table 14). The CBS(2-4) estimate for Benzene dimer is 2.66 kcal/mol which is already consistent with the existing result [14, 15]. Leveraging this fact, we are able to calculate the Benzene dimer CBS(3-5) result with Benzene monomer CBS(3-5) result and binding energy (2-4) result, which gives -464.479015 Ha. Namely $E_{4.95\ (3-5)} = 2 \times E_{\mathrm{mono}\ (3-5)} - E_{\mathrm{binding}\ (2-4)}$. We believe this is more reliable

Supplementary Table 14 | Extrapolation of CBS limit for binding energy of benzene dimer

basis	CCSD(T) binding energy	
	(hartree)	(kcal/mol)
cc-pCVDZ	0.004855	3.046686
cc-pCVTZ	0.004659	2.923544
cc-pCVQZ	0.004526	2.839844
CBS(2-4)	0.004243	2.662232

than the CBS(2-4) result and report it in the main text Figure 5a. Here we don't consider the Basis Set Superposition Error (BSSE) because the difference between uncorrected and BSSE-corrected CBS estimates is negligible, according to [14].

Supplementary Note 15. Details on the divergence measuring nodal surface difference

The neighborhood S_ϵ and mapping ϕ introduced in main text Section IV F for the wavefunction Ψ and nodal surface S is difficult to compute in practice. Instead we propose easier-to-compute alternatives as approximation.

The major difficulty here is that given a point y and a set S, it's hard to find the point $z \in S$ that is closest to y. We propose an approximation as follows. Note that the set S of our interest is always a nodal surface associated with certain wavefunction Ψ. Instead of looking for $z \in S$ that is closest to y, we move y in the direction in which the value of $\log(|\Psi|)$ decreases the fastest, namely we form the integral curve of the vector field $-\nabla \log(|\Psi|)$ starting from y. And we use the intersection of this integral curve and S as the target point z, signaled by the change of sign of Ψ, and we approximate the distance from y to S as

$$\tilde{d}(y, S) = d(y, z)$$

In other words, we are looking for $z \in S$ such that there exists curve $l(t)$ satisfying

$$\begin{cases} \frac{dl}{dt} = -\nabla \log(|\Psi(l)|) \\ l(0) = y \\ l(t_0) = z \in S \end{cases}$$

where t_0 could be an arbitrary number, either positive or negative. Denote the function mapping y to $z \in S$ as ξ, and ξ is used as our practical substitute for function ϕ mentioned in main text Section IV F looking for the closest point to y in S. Function ξ is well-defined in the neighbourhood of S, guaranteed by the local existence and uniqueness of ODE.

Similarly, we propose the alternative of $S_\epsilon = \{x | d(x, S) < \epsilon\}$ as

$$\tilde{S}_\epsilon = \{x | \Psi(x) \cdot \Psi(x - \epsilon \cdot \frac{\nabla \log(|\Psi(x|)}{||\nabla \log(|\Psi(x)|)||}) < 0\}$$

where S is the nodal surface for wavefunction Ψ.

Our algorithm to compute the approximated divergence is in Supplementary Algo. 1.

Supplementary Algorithm 1: Divergence for nodal surface difference pseudocode.

Data: wavefunction Ψ for nodal surface S. wavefunction Φ for nodal surface T. A batch of walkers (W) following the distribution of normalized Ψ^2. The number of MCMC steps (M) to run in each iteration. ϵ controlling the size of neighborhood of nodal surfaces. η controlling the discretization of integral curve when moving towards the nodal surface.

Output: Divergence value D measuring difference between S and T.

Initialize set L as an empty set. ▷ Sample points from S_ϵ according to Ψ^2
while *length(L) < K* **do**
  for *each walker r_i in W* **do**
    Run MCMC procedure from r_i for M steps to get a new walker q_i.
    $u_i = q_i - \epsilon \cdot \frac{\nabla \log(|\Psi(q_i)|)}{||\nabla \log(|\Psi(q_i)|)||}$
    if $\Psi(u_i) \cdot \Psi(q_i) < 0$ **then**
      Insert q_i into L
    end
    $r_i \leftarrow q_i$
  end
end

Initialize set O as an empty set. ▷ Move samples in L closer to node Surface S of Ψ
for *each walker in L* **do**
  Move along the vector field $\frac{\nabla \log(|\Psi|)}{||\nabla \log(|\Psi|)||}$ with tiny step η until the sign of Ψ changes
  $o_i \leftarrow$ the point right before the sign changes
  Insert o_i into O
end
▷ Move samples in O closer to node surface T of Φ
for *each walker o_i in O* **do**
  Move along the vector field $\frac{\nabla \log(|\Phi|)}{||\nabla \log(|\Phi|)||}$ with tiny step η until the sign of Φ changes
  $p_i \leftarrow$ the point right before the sign changes
  compute $d(o_i, p_i)$
end
compute D = Avg($d(o_i, p_i)$)

Supplementary References

[1] Richard Needs, Mike Towler, Neil Drummond, and Pablo López Rıos. User's guide version 2.13 (2019), 2019.

[2] Henrik Flyvbjerg and Henrik Gordon Petersen. Error estimates on averages of correlated data. *The Journal of Chemical Physics*, 91(1):461–466, 1989.

[3] RJ Needs, MD Towler, ND Drummond, Pablo Lopez Rios, and JR Trail. Variational and diffusion quantum monte carlo calculations with the casino code. *The Journal of chemical physics*, 152(15):154106, 2020.

[4] Andrea Zen, Sandro Sorella, Michael J Gillan, Angelos Michaelides, and Dario Alfe. Boosting the accuracy and speed of quantum monte carlo: Size consistency and time step. *Physical Review B*, 93(24):241118, 2016.

[5] D. Pfau, J.S. Spencer, A.G. de G. Matthews, and W.M.C. Foulkes. Ab-initio solution of the many-electron schrödinger equation with deep neural networks. *Phys. Rev. Research*, 2:033429, 2020.

[6] James S. Spencer, David Pfau, Aleksandar Botev, and W. M. C. Foulkes. Better, Faster Fermionic Neural Networks. *arXiv:2011.07125 [physics]*, November 2020. arXiv: 2011.07125.

[7] James Spencer and David Pfau. FermiNet: Fermionic Neural Networks, 2020.

[8] James Martens and Roger Grosse. Optimizing neural networks with kronecker-factored approximate curvature. In *International conference on machine learning*, pages 2408–2417. PMLR, 2015.

[9] Aleksandar Botev and James Martens. KFAC-JAX, 2022.

[10] Jeffmin Lin, Gil Goldshlager, and Lin Lin. Explicitly antisymmetrized neural network layers for variational monte carlo simulation, 2021.

[11] Arne Lüchow and James B Anderson. Accurate quantum monte carlo calculations for hydrogen fluoride and the fluorine atom. *The Journal of chemical physics*, 105(11):4636–4640, 1996.

[12] Daniel G. A. Smith et al. Psi4 1.4: Open-source software for high-throughput quantum chemistry. *The Journal of Chemical Physics*, 152(18):184108, 2020.

[13] Asger Halkier, Trygve Helgaker, Poul Jørgensen, Wim Klopper, and Jeppe Olsen. Basis-set convergence of the energy in molecular hartree–fock calculations. *Chemical Physics Letters*, 302(5-6):437–446, 1999.

[14] Evangelos Miliordos, Edoardo Aprà, and Sotiris S Xantheas. Benchmark theoretical study of the π–π binding energy in the benzene dimer. *The Journal of Physical Chemistry A*, 118(35):7568–7578, 2014.

[15] J. R. Grover, E. A. Walters, and E. T. Hui. Dissociation energies of the benzene dimer and dimer cation. *The Journal of Physical Chemistry*, 91(12):3233–3237, 1987.
